# NADH‐Reductive Stress Induced by Dihydrolipoamide Dehydrogenase Activation Contributes to Cuproptosis

**DOI:** 10.1002/advs.202520444

**Published:** 2025-12-05

**Authors:** Si‐Yi Zhang, Xing‐Hua Ren, Cheng‐Hong Zhang, Zhan‐You Wang

**Affiliations:** ^1^ Key Laboratory of Medical Cell Biology of Ministry of Education Key Laboratory of Major Chronic Diseases of Nervous System of Liaoning Province Health Sciences Institute of China Medical University Shenyang 110122 China; ^2^ School of Forensic Medicine China Medical University Shenyang 110122 China; ^3^ Basic Medical Experimental Teaching Center China Medical University Shenyang 110122 China

**Keywords:** copper, cuproptosis, dihydrolipoamide dehydrogenase, mitochondrial permeability transition pore, NADH‐reductive stress

## Abstract

Copper (Cu) is an essential trace element for cellular metabolism, while excessive Cu accumulation leads to neurotoxicity. Current therapeutic strategies for Cu overload remain inadequate in mitigating neurological symptoms. The recently discovered Cu‐dependent mitochondrial cell death pathway, cuproptosis, offers novel insights into Cu‐mediated neurotoxicity. In this study, the mechanistic link between mitochondrial respiration and cuproptosis is elucidated. The current study demonstrates that activated dihydrolipoamide dehydrogenase (DLD), induced by excess Cu under alkaline mitochondrial pH conditions, drives nicotinamide adenine dinucleotide (NADH) accumulation. Cu mediated mitochondrial permeability transition pore (mPTP) opening that facilitates NADH translocation to the cytosol, triggering NADH‐reductive stress. This promotes aberrant purine biosynthesis, leading to severe adenosine triphosphate depletion and energy stress. Pharmacological interventions targeting DLD activity, cytosolic NADH, mPTP opening, purine biosynthesis, or energy stress effectively rescued Cu‐induced cell death in SH‐SY5Y neuroblastoma cells. Collectively, these findings reveal characteristics of NADH‐reductive stress under excessive Cu exposure, establishing cuproptosis as a novel NADH‐reductive stress‐dependent cell death pathway. This mechanistic insight provides new therapeutic avenues for Cu‐associated neurological pathologies and new aspects to explore Cu cellular physiology.

## Graphical abstract

This study demonstrates a cuproptosis mechanism involving nicotinamide adenine dinucleotide (NADH)‐reductive stress in neural cells. Copper activates dihydrolipoamide dehydrogenase under mitochondrial pH, accumulating NADH. Copper also induces mitochondrial permeability transition pore opening, facilitating NADH translocation to the cytosol and provoking reductive stress, aberrant purine biosynthesis, and adenosine triphosphate depletion, resulting in cell death. Targeting the NADH‐reductive stress pathway offers a novel therapeutic strategy for copper‐associated neurodegeneration.

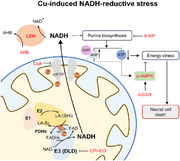

## 1 Introduction

Copper (Cu), acting as a co‐factor for numerous metabolic enzymes for its redox‐active feature, plays a critical role in various cellular processes, including energy metabolism and antioxidant defense. However, excessive Cu accumulation can lead to neurological impairments.^[^ ^1^ ^]^ In a Cu‐associated disorder, Wilson's disease (WD), psychiatric and cognitive symptoms are common.^[^ ^2^ ^]^ Current therapeutic strategies for WD primarily involve Cu chelation and oral zinc supplementation. Notably, some patients exhibit neurological worsening when initiating treatment of WD, demonstrating the limitation of existing therapeutic approaches.^[^ ^3^ ^]^ Accumulating evidence from multiple studies has established a significant association between Cu and neurodegenerative disorders, such as Alzheimer's disease and Parkinson's disease.^[^ ^4^, ^5^ ^]^ Additionally, a recent study identified elevated hippocampal Cu levels in cases of type 2 diabetes, potentially contributing to cognitive dysfunction.^[^ ^6^ ^]^ These observations highlight the need for a deeper understanding of the pathological mechanisms underlying Cu toxicity in neural cells, which remain poorly elucidated.

**Figure 1:**
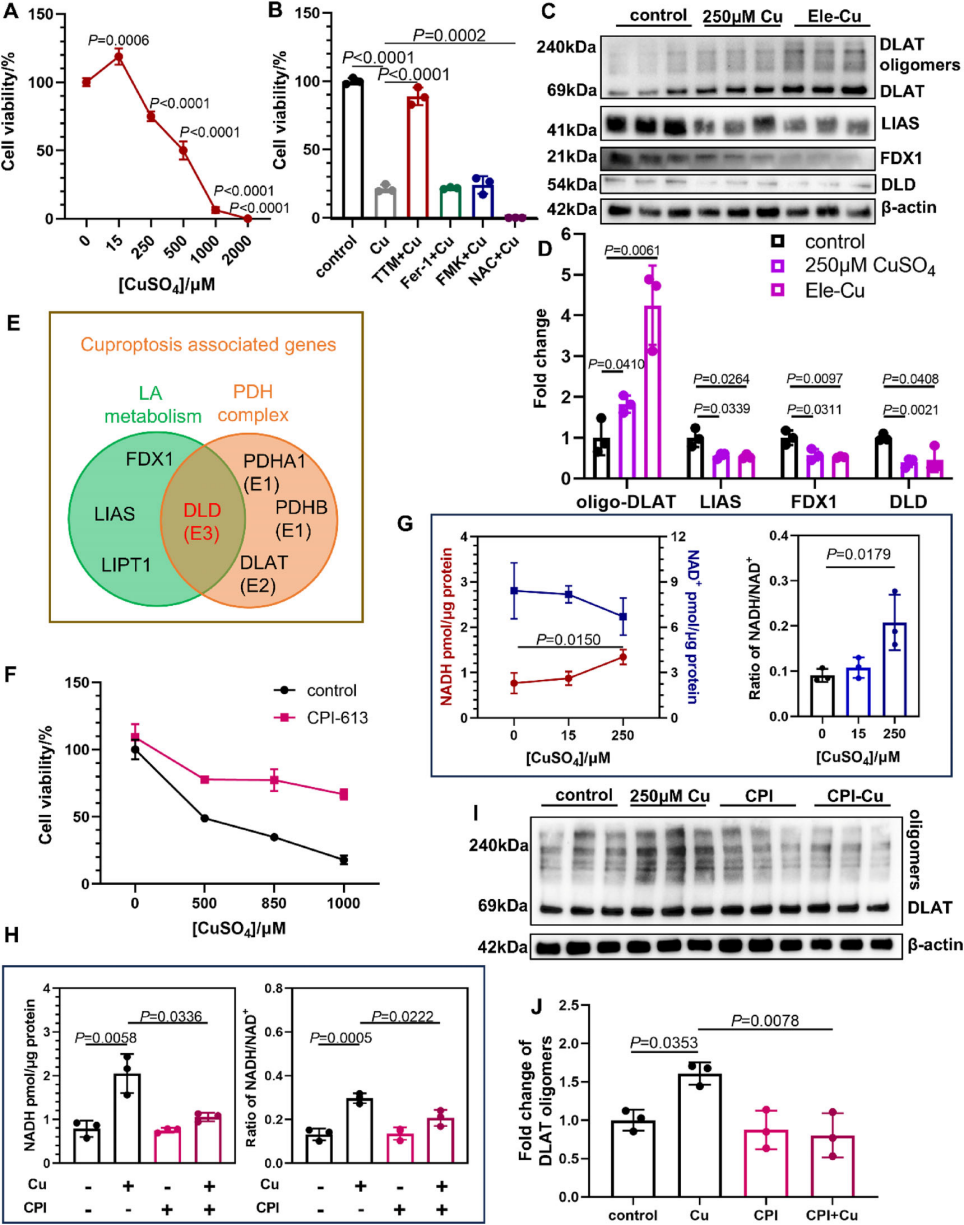
CuSO_4_‐induced cuproptosis and NADH accumulation can be attenuated by DLD inhibition. A) Viability of SH‐SY5Y cells after 24 h treatment with CuSO_4_ (*n* = 3, one‐way ANOVA with Dunnett's test). B) Viability of cells pretreated 2 h with 5 µg mL^−1^ ammonium tetrathiomolybdate (TTM), 10 µm ferrostatin‐1(Fer‐1), 50 µm Boc‐D‐FMK (FMK), 5 mm N‐acetylcysteine (NAC), then treated with 1 mm CuSO_4_ for 18 h (*n* = 3, unpaired *t*‐test). C,D) Western blot analysis (C) and quantifications (D) reflecting cuproptosis of SH‐SY5Y cells treated with 250 µm CuSO_4_ or 50 nm elesclomol‐ 50 µm CuSO_4_ pulse (2 h) treatment for 24 h (*n* = 3, unpaired *t*‐test). E) Cuproptosis‐associated genes participate in either lipoic acid (LA) metabolism or the pyruvate dehydrogenase complex (PDHc). FDX1, ferredoxin1. LIAS, lipoyl synthase. LIPT1, lipolytransferase1. DLD, dihydrolipoamide dehydrogenase, PDHc E3 subunit. PDHA1, pyruvate dehydrogenase E1 subunit alpha 1, PDHc E1 subunit. PDHB, pyruvate dehydrogenase E1 subunit beta, PDHc E1 subunit. DLAT, dihydrolipoamide S‐acetyltransferase, PDHc E2 subunit. F) Viability of SH‐SY5Y cells treated with CuSO_4_ and 4 nm CPI‐613 for 18 h (*n* = 3). G) NADH level and NADH/NAD^+^ ratio of SH‐SY5Y cells after CuSO_4_ treatment for 18 h (*n* = 3, one‐way ANOVA with Dunnett's test). H) NADH level and NADH/NAD^+^ ratio of SH‐SY5Y cells treated with 250 µm CuSO_4_ and 4 nm CPI‐613 (CPI) for 18 h (*n* = 3, two‐way ANOVA with Tukey's test). I,J) Western blot analysis (I) and quantifications (J) of DLAT oligomerization in SH‐SY5Y cells treated with 250 µm CuSO_4_ and 4 nm CPI‐613(CPI) for 24 h (*n* = 3, two‐way ANOVA with Tukey's test). Means ± SD. Images of the cell viability assay are shown in the Supplementary Information.

Cuproptosis, a recently discovered form of regulated cell death induced by mitochondrial Cu accumulation, offers novel insights into Cu‐related pathology. Cuproptosis is a Cu‐dependent, mitochondrially induced cell death, demonstrating distinct characteristics from apoptosis, ferroptosis, and necroptosis, and occurs independently of oxidative stress.^[^ ^7^ ^]^ Current research indicates that mitochondrial respiration serves as a fundamental requirement for cuproptosis. A total of 7 identified cuproptosis‐associated genes all encode either components of the lipoic acid (LA) metabolism or the pyruvate dehydrogenase complex (PDHc) (Figure 1E), which are both essential for mitochondrial respiration.^[^ ^7^, ^8^ ^]^ Tsvetkov et al. have elucidated the critical role of protein lipoylation in cuproptosis, demonstrating that inhibition of mitochondrial pyruvate carrier and electron transport chain attenuates Cu‐induced cell death, thereby substantiating the crucial involvement of PDHc and mitochondrial respiration in cuproptosis.^[^ ^7^ ^]^

However, the underlying molecular mechanisms through which PDHc and mitochondrial respiration contribute to cuproptosis remain to be fully elucidated. PDHc catalyzes the rate‐limiting step of mitochondrial respiration in eukaryocytes, mediating the crucial conversion of pyruvate to acetyl‐coenzyme A while generating nicotinamide adenine dinucleotide (NADH), connecting glycolysis and the tricarboxylic acid (TCA) cycle.^[^ ^9^ ^]^ Structurally, PDHc comprises three essential enzymatic subunits, including E1, E2, and E3, all of which are encoded by cuproptosis‐associated genes. During PDHc catalysis, the E3 subunit dihydrolipoamide dehydrogenase (DLD) oxidizes dihydrolipoamide on the E2 subunit dihydrolipoamide S‐acetyltransferase (DLAT) using flavin adenine dinucleotide (FAD) as a cofactor, thereby regenerating the oxidized lipoyl moiety for subsequent catalytic cycles. Finally, DLD transfers electrons from the reduced form of FAD (FADH_2_) to the oxidized form of NADH (NAD^+^), producing NADH while restoring FAD.^[^ ^8^ ^]^ Interestingly, DLD functions not only in PDHc, but it is also an essential component of other lipoic acid‐dependent multienzyme complexes in the mitochondrial matrix, including α‐ketoglutarate dehydrogenase complex involved in the TCA cycle.^[^ ^8^ ^]^ Consequently, DLD plays an essential role in NADH production and mitochondrial respiration.

It is well‐established that NADH provides electrons for mitochondrial oxidative phosphorylation, driving adenosine triphosphate (ATP) production to sustain cellular viability. However, pathological accumulation of cytosolic NADH can induce NADH‐reductive stress associated with cell death.^[^ ^10^ ^]^ Previous studies revealed that Cu facilitates the opening of mitochondrial permeability transition pore (mPTP), a non‐specific channel at the inner mitochondrial membrane.^[^ ^11^, ^12^ ^]^ The mPTP opening enables NADH to translocate from the mitochondrial matrix to the cytosol, which may induce NADH‐reductive stress.^[^ ^13^ ^]^ Cytosolic NADH‐reductive stress is characterized by an elevated NADH/NAD^+^ ratio and can be alleviated through lactate dehydrogenase (LDH)‐mediated conversion of pyruvate to lactate coupled with NADH oxidation.^[^ ^10^ ^]^ The recent work by Yang et al. has uncovered a novel mechanistic pathway of NADH‐reductive stress. Their findings demonstrated that accumulated NADH directly interacts with phosphoribosyl pyrophosphate synthetase 2 (PRPS2), disrupting ADP‐dependent negative feedback regulation. This interaction leads to severe ATP depletion through provoked purine biosynthesis and ultimately triggers energy stress‐induced cell death.^[^ ^14^, ^15^, ^16^ ^]^

In the current study, we applied copper sulfate (CuSO_4_) to human neuroblastoma SH‐SY5Y cells, exploring the role of NADH‐reductive stress in Cu neurotoxicity. Our study specifically addressed whether Cu exposure modulates DLD activity to promote cytosolic NADH accumulation, and sought to establish the potential mechanistic link between NADH‐reductive stress and cuproptosis in neural cells. Our findings demonstrate a Cu‐dependent upregulation of DLD activity that exhibited pH sensitivity. Furthermore, we established that pathological accumulation of cytosolic NADH, resulting from DLD activation and Cu‐induced mPTP opening, indeed induces NADH‐reductive stress. Importantly, pharmacological targeting of both DLD activity and NADH‐reductive stress effectively attenuated Cu‐induced cell death. These results collectively identify cuproptosis as a novel form of NADH‐reductive stress‐dependent cell death, providing critical insights into Cu homeostasis and pathology in neurological contexts.

## 2 Results

### 2.1 Cu‐Induced Cell Death and NADH Accumulation were Mediated by DLD in SH‐SY5Y Cells

In this study, we employed CuSO_4_ to induce cuproptosis in SH‐SY5Y neuroblastoma cells. Cell viability assessment using Calcein AM/propidium iodide (PI)/Hoechst triple staining revealed a significant dose‐dependent reduction in viability at CuSO_4_ concentrations of 250 µm and above (**Figure** 1A; Figure S1, Supporting Information). Notably, the cell death induced by CuSO_4_ was specifically rescued by the Cu chelator ammonium tetrathiomolybdate (TTM), but not by the pan‐caspase inhibitor Boc‐D‐FMK (FMK), ferroptosis inhibitor ferrostatin‐1 (Fer‐1), or antioxidant N‐acetylcysteine (NAC) (Figure 1B; Figure S2, Supporting Information), consistent with the rescue pattern observed in Cu ionophore elesclomol‐Cu treatment (Figure S3, Supporting Information). Molecular characterization demonstrated representative biomarkers of cuproptosis following both 250 µm CuSO_4_ treatment and elesclomol‐Cu pulse exposure, including downregulation of Fe‐S cluster protein ferredoxin1(FDX1) and lipoyl synthase (LIAS) levels, along with DLAT oligomerization (Figure 1C,D).^[^ ^7^ ^]^ Moreover, a downregulated DLD level was also observed in both treatments (Figure 1C,D). These collective findings establish CuSO_4_ as an effective inducer of cuproptosis in this cellular model.

To further elucidate the molecular mechanisms underlying cuproptosis, we analyzed the genome‐wide CRISPR screening data of both Cu ionophores (elesclomol and diethyldithiocarbamate) from the work of Tsvetkov et al. by applying stringent selection criteria of log Fold Change >1 and false discovery rate < 0.01.^[^ ^7^ ^]^ This analysis identified *FDX1*, *LIAS*, and *DLD* as significant contributors to the cuproptosis process. FDX1 and LIAS participate in lipoic metabolism, whereas DLD serves dual roles in both lipoic acid metabolism and the PDHc (Figure 1E). To investigate the contribution of DLD to cuproptosis, cells were treated with 250 µm CuSO_4_ in combination with CPI‐613, a lipoic acid analog and specific DLD inhibitor.^[^ ^17^ ^]^ The results showed that CPI‐613 significantly rescued Cu‐induced cell death (Figure 1F; Figure S4, Supporting Information), demonstrating that DLD enzymatic activity is essential for cuproptosis.

Biologically, DLD catalyzes the regeneration of oxidized lipoic acid while concomitantly generating NADH.^[^ ^8^ ^]^ Tsvetkov et al. demonstrated that FDX1 knockout leads to significant NADH depletion, confirming the crucial role of lipoic acid metabolism in maintaining cellular NADH pools.^[^ ^7^ ^]^ These findings suggest that DLD may serve as a key effector in cuproptosis by driving mitochondrial respiration and NADH accumulation during Cu exposure. First, we quantitatively measured both NADH levels and the NADH/NAD^+^ ratio in SH‐SY5Y cells following 18 h CuSO_4_ exposure. As expected, a high dose of 250 µm CuSO_4_ treatment significantly induced intracellular NADH accumulation (Figure 1G). Then, CPI‐613 was used to pharmacologically inhibit DLD function. Consistent with our prediction, CPI‐613 effectively blocked Cu‐induced elevation of NADH levels and the NADH/NAD^+^ ratio (Figure 1H), providing direct evidence that DLD mediates NADH accumulation during Cu exposure in neural cells.

Given that DLD catalyzes disulfide bond formation in the lipoyl moiety of DLAT, we investigated whether Cu‐induced DLAT oligomerization, which is also disulfide bond‐dependent, is mediated by DLD activity.^[^ ^7^, ^8^ ^]^ Consistent with this hypothesis, CPI‐613 treatment significantly attenuated Cu‐induced DLAT oligomerization (Figure 1I,J). These data provide compelling evidence that DLD enzymatic activity constitutes a critical role in the cuproptosis pathway, and excessive Cu induces functional enhancement of DLD independent of its protein abundance.

### 2.2 DLD was Activated by Cu Through Disulfide Bond Formation

**Figure 2:**
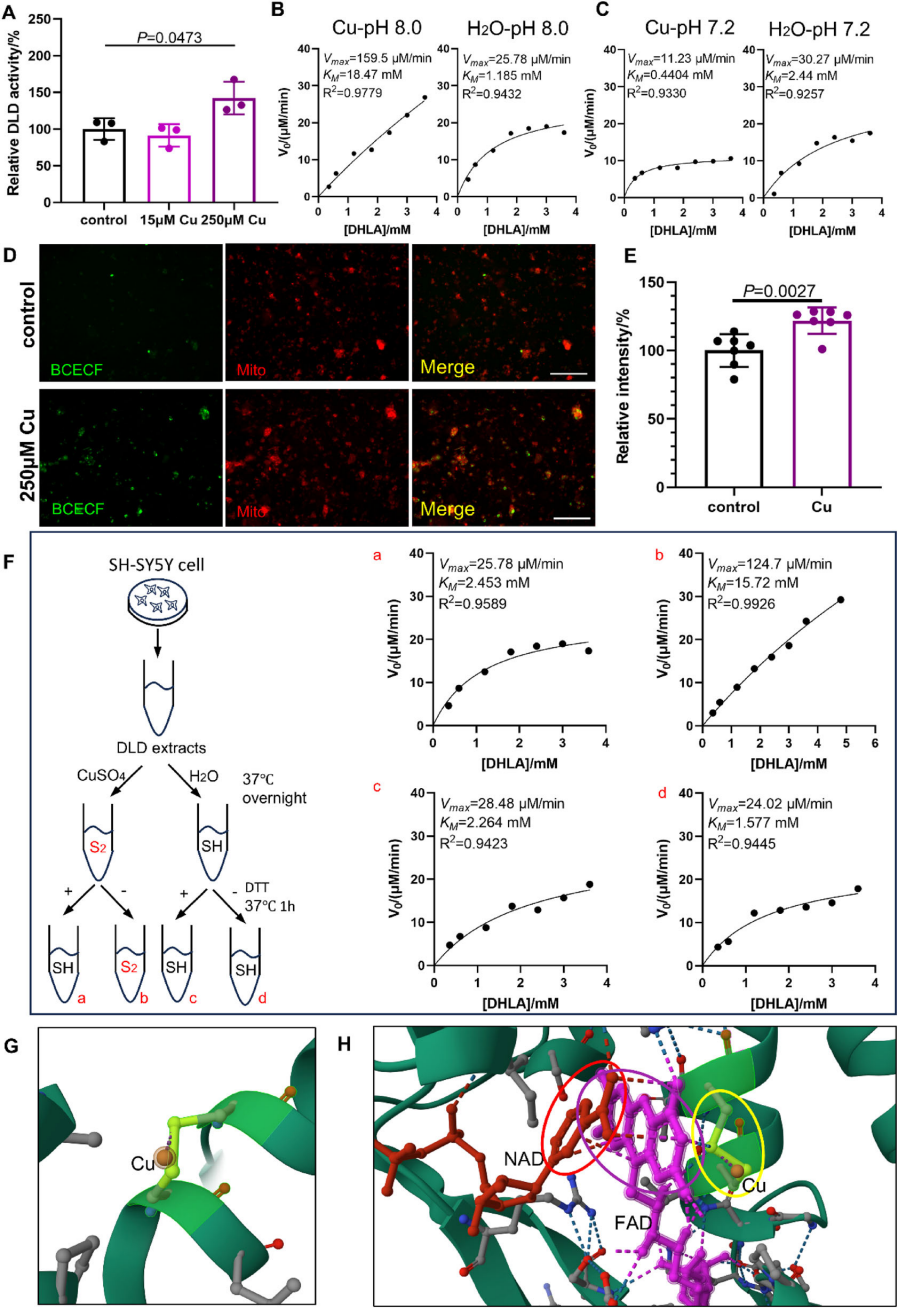
Cu activated mitochondrial DLD through disulfate bond formation. A) DLD activity of SH‐SY5Y cells after 24 h CuSO_4_ treatment measured at pH 8.0 (*n* = 3, one‐way ANOVA with Dunnett's test). B,C) DLD kinetic assay at pH *8.0* (B) or *7.2* (C) of cell extracts incubated with CuSO_4_ or H_2_O overnight. D,E) The image (D) and quantifications (E) of isolated mitochondria pH probed using BCECF‐AM(green) and Mito‐Tracker Red CMXRos (Mito, red) after 24 h treatment with or without 250 µm CuSO_4_ (*n* = 7, unpaired *t*‐test). Scale bar, 100 µm. Means ± SD. F)The workflow (left) and plots (a–d) of DLD enzyme kinetics using dihydrolipoic acid (DHLA) as the substrate after incubation of CuSO_4_ and DTT. S_2_, disulfate bond. SH, free thiol group. G) The predicted interaction between Cu ion (Bronze‐colored small ball) and DLD Cys80 and Cys85 (highlighted) generated by AlphaFill. The yellow‐labeled covalent bond indicated a disulfate bond between Cys80 and Cys85. H) Interaction among DLD cofactor FAD (purple molecule), NAD (red molecule), and DLD Cys80 and Cys85 residues (highlighted) generated by AlphaFill. The structures marked with red, purple, and yellow circles are nicotinamide in NAD, the isoalloxazine ring of the FAD, and the disulfate bond between Cys80 and Cys85 residues of DLD, respectively. The bronze‐colored small ball indicates Cu ion. The dashed lines indicate a hydrogen bond or salt bridge between the indicated atoms.

To further confirm DLD activation during Cu exposure, the enzymatic activity of DLD from SH‐SY5Y cells was tested. Given the physiological pH *8.0* of the mitochondrial matrix where DLD normally functions, we assessed the activity of DLD oxidizing dihydrolipoic acid (DHLA) at this optimal pH by monitoring NADH production spectrophotometrically at 340 nm.^[^ ^18^, ^19^, ^20^ ^]^ Results showed that 24 h exposure to 250 µm CuSO_4_ significantly enhanced DLD enzymatic activity in SH‐SY5Y cells compared to untreated controls (**Figure** **2**A). Notably, while multiple studies report Cu‐induced suppression of DLD activity, these observations were made at sub‐physiological pH conditions (≤7.2).^[^ ^21^, ^22^ ^]^ To evaluate the pH‐dependence of Cu's effects on DLD activity, the DLD extracts of SH‐SY5Y cells were incubated with either CuSO_4_ or vehicle control (H_2_O) overnight at 37 °C, followed by enzymatic kinetic assays at pH *7.2* and *8.0*. DLD after Cu pretreatment provided higher maximum reaction velocity (*V_max_*) at pH *8.0* compared to controls, while exhibiting the expected lower *V_max_* at pH *7.2* (Figure 2B,C). These results indicate a significant impact of pH on DLD activity after Cu treatment and demonstrate that the alkaline mitochondrial environment is essential for Cu‐mediated DLD activation. Given reports of Cu‐induced mitochondria pH alteration in different cell types, we employed the pH‐sensitive probe BCECF‐AM to detect pH changes in both whole cells and isolated mitochondria following 24 h CuSO_4_ treatment.^[^ ^23^, ^24^, ^25^ ^]^ While 250 µm CuSO_4_ in culture medium did not interfere with the BCECF fluorescence (Figure S5A, Supporting Information), the observed increase in BCECF fluorescence intensity confirmed Cu‐induced alkalinization at both cellular (Figure S5B,C, Supporting Information) and mitochondrial levels (Figure 2D,E). This provides a mechanistic basis for the observed Cu‐dependent DLD activation in neural cells.

Previous studies have proposed that Cu‐induced modulation of DLD activity may involve irreversible structural modifications mediated by disulfide bond formation.^[^ ^22^ ^]^ To validate this mechanism, we performed kinetic analyses of DLD enzymatic activity following Cu exposure. The Michaelis‐Menten constant (𝐾_𝑀_) and maximum reaction velocity (*V_max_*) were determined with or without reduction of potential disulfide bonds using dithiothreitol (DTT). Notably, Cu‐treated DLD exhibited significantly elevated 𝐾_𝑀_ and *V_max_* values compared to native enzyme, and these kinetic alterations were reversed by DTT treatment (Figure 2F). These findings demonstrate that Cu induces disulfide bond‐dependent conformational changes in DLD that simultaneously modulate its substrate affinity and catalytic capacity.

We applied an artificial intelligence‐based protein structure prediction approach AlphaFill, to identify potential Cu‐binding sites that could facilitate disulfide bond formation in DLD.^[^ ^26^ ^]^ Our analysis revealed 29 potential Cu‐DLD binding sites. Among them, a putative Cu‐binding site involving Cys80 and Cys85 potentially forms a disulfide bond with the following structural parameters: global root‐mean‐square deviation (r.m.s.d.) = 4.85 Å, local r.m.s.d. = 0.31 Å, transplant clash score (TCS) = 0.76 Å (Figure 2G). Moreover, consistent with a previous study, the isoalloxazine ring of the FAD undergoing proton exchange interacts with DLD Cys80 and Cys85 in the AlphaFill predicted structure.^[^ ^27^ ^]^ Also, the nicotinamide in NAD establishes direct interaction with the isoalloxazine ring of the FAD (Figure 2H), suggesting a potential electron transfer pathway between these cofactors. These findings suggest a possible mechanism that Cu may allosterically activate mitochondrial DLD through disulfide bond formation between Cys80 and Cys85 residues, potentially modulating the interaction of the enzyme with its essential cofactors.

### 2.3 Upregulated NADH was Localized to the Cytosol Through Cu‐Mediated mPTP Opening

**Figure 3:**
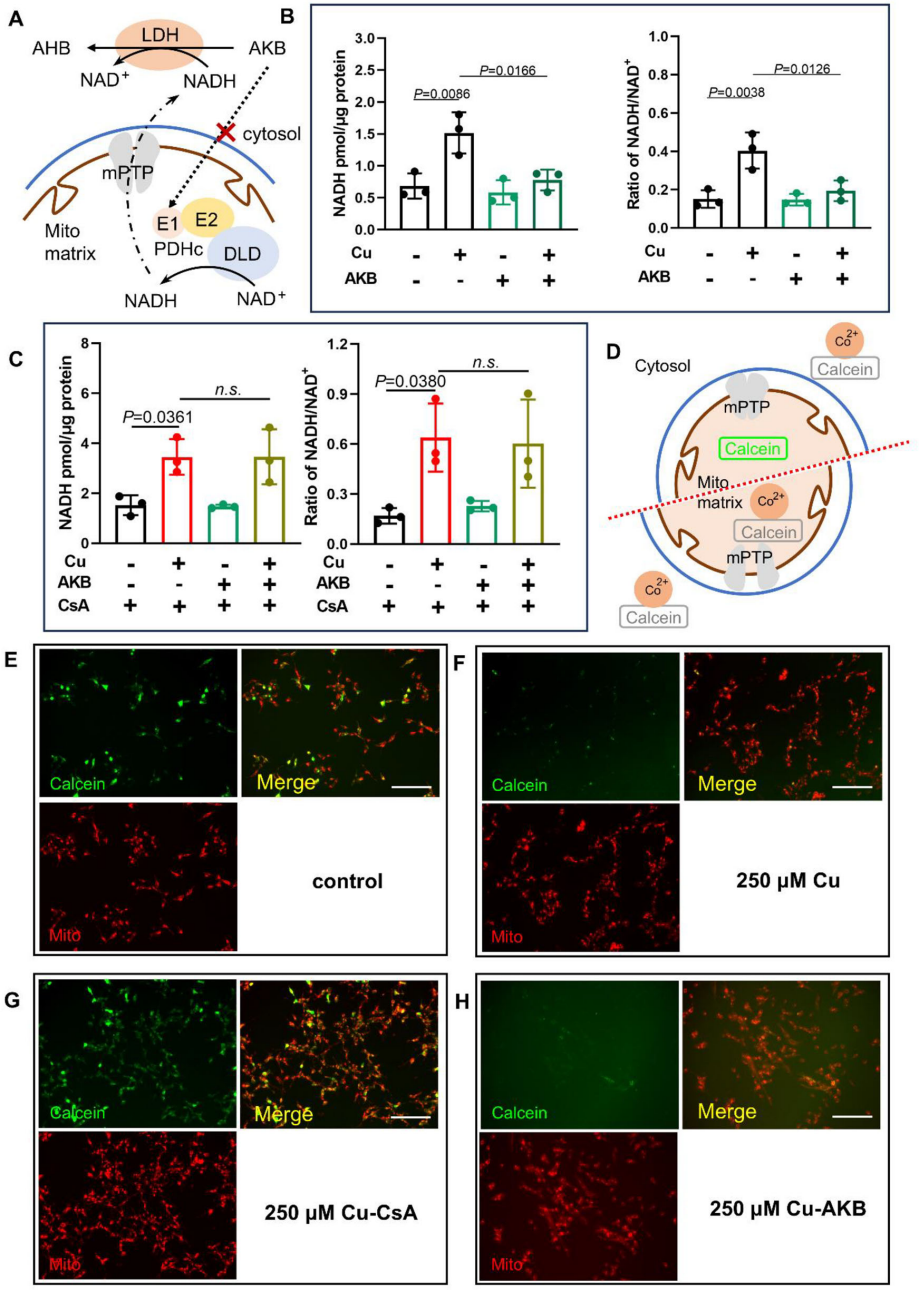
NADH translocated to the cytosol through Cu‐mediated mPTP opening. A) Schematic of AKB converses cytosolic NADH accumulation related to DLD. AKB, α‐ketobutyrate. AHB, α‐hydroxybutyrate. mPTP, mitochondrial permeability transition pore. B) The NADH level and NADH/NAD^+^ ratio in SH‐SY5Y cells after 18 h treatment with 250 µm CuSO_4_ and 1 mm AKB (*n* = 3, two‐way ANOVA with Tukey's test). C) The NADH level and NADH/NAD^+^ ratio in SH‐SY5Y cells from 18 h treatment with 250 µm CuSO_4_ and 1 mm AKB after 2 h CsA pretreatment (*n* = 3, two‐way ANOVA with Tukey's test). Means ± SD. D) Schematic of mPTP opening assay using Calcein AM fluorescence quenching assay. Calcein fluorescence (green) is diffusely distributed in the cells, while cobalt ions (Co^2+^) can quench the Calcein fluorescence. Co^2+^ is not allowed to enter the mitochondrial matrix unless the mPTP is in an open state. The quenching of mitochondrial Calcein fluorescence indicates the opening of mPTP. E,H). The mPTP state after 24 h treatment of vehicle control (E), 250 µm CuSO_4_ without CsA pretreatment F), 250 µm CuSO_4_ with CsA pretreatment G), and 250 µm CuSO_4_ with 1 mm AKB (H). Calcein fluorescence (green), Mito‐Tracker Red CMXRos (Mito, red). Scale bar, 100 µm.

Subsequently, we investigated whether Cu‐induced NADH accumulation exhibited pathological cytosolic localization that could potentially trigger NADH‐reductive stress. We employed a pyruvate analog, α‐ketobutyrate (AKB), that serves exclusively as an electron acceptor in the cytosol to convert the cytosolic NADH in cells. The AKB converts NADH to NAD^+^ through LDH while producing the excretory metabolite α‐hydroxybutyrate (AHB), without directly participating in carbon metabolism (**Figure** **3**A).^[^ ^28^ ^]^ Our results demonstrate that AKB treatment effectively normalized Cu‐induced NADH accumulation to levels comparable with control conditions (Figure 3B). This finding suggests that Cu exposure leads to pathological elevation of cytosolic NADH, which is sufficient to induce NADH‐reductive stress.

Under physiological conditions, NADH cannot freely traverse the mitochondrial inner membrane. While NADH produced during glycolysis can be shuttled to the mitochondrial matrix via established shuttle systems (including malate/aspartate shuttle and the glycerol‐3 phosphate shuttle), no specific transport mechanism has been identified for the reverse translocation of NADH from the mitochondrial matrix to the cytosol, except for the mPTP‐mediated leakage.^[^ ^13^ ^]^ To investigate whether Cu‐induced elevation of cytosolic NADH is mediated by mPTP opening, we pretreated cells with cyclosporin A (CsA), a well‐characterized mPTP inhibitor, before Cu exposure. Consistent with our hypothesis, the inhibition of mPTP by CsA abrogated the ability of AKB to reduce Cu‐induced NADH accumulation (Figure 3C).^[^ ^29^ ^]^ Therefore, although direct visualization is lacking, our results supported the conclusion that mPTP opening is essential for the observed NADH distribution to the cytosol.

The mPTP state was assessed using the Calcein AM fluorescence quenching assay. This method relies on the cobalt ion (Co^2+^)‐mediated quenching of mitochondrial Calcein fluorescence, which occurs exclusively when mPTP is in the open state (Figure 3D). Our results demonstrated a significant loss of Calcein fluorescence co‐localization with a mitochondria‐specific probe following treatment with 250 µm CuSO_4_ (Figure 3E,F), whereas no such effect was observed after 15 µm CuSO_4_ treatment (Figure S6, Supporting Information), confirming dose‐dependent mPTP opening by Cu. Importantly, treatment with CsA, but not AKB, effectively prevented Cu‐induced mPTP opening (Figure 3G,H), indicating that AKB did not interfere with mPTP state, and NADH accumulation was not necessary for Cu‐triggered mPTP opening.

### 2.4 NADH‐Reductive Stress Induces Cuproptosis

**Figure 4:**
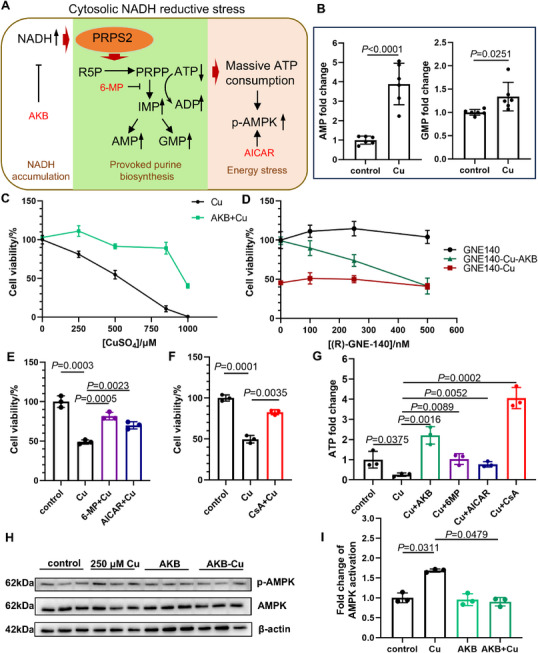
Excessive Cu induced cell death through the NADH‐reductive stress‐related pathway. A) Schematic of cytosolic NADH‐reductive stress involving purine biosynthesis and energy stress. NADH interacts with PRPS2 to provoke purine biosynthesis, which causes huge ATP consumption and induces energy stress. The upregulated phosphorylation of AMPK is a self‐defense response of cells under energy stress. Compounds marked in red are protective against NADH‐reductive stress. PRPS2, phosphoribosyl pyrophosphate synthetase 2. R5P, Ribose 5‐phosphate. PRPP, phosphoribosyl pyrophosphate. p‐AMPK, phosphorylated AMP‐activated protein kinase. AKB, α‐ketobutyrate. 6‐MP, 6‐mercaptopurine. AICAR, 5‐aminoimidazole‐4‐carboxamide ribonucleoside. B) AMP and GMP levels of SH‐SY5Y cells treated with 250 µm CuSO_4_ for 24 h (unpaired *t*‐test, *n* = 6). C) Viability of SH‐SY5Y cells after 24 h treatment with 1 mm AKB and CuSO_4_. n = 3. D) Viability of SH‐SY5Y cells after 24 h treatment with 1 mm AKB, 500 µm CuSO_4,_ and (R)‐GNE‐140. *n* = 3. E) Viability of SH‐SY5Y cells treated with 500 µm CuSO_4_ and 100 µm 6‐MP, 400 µm AICAR for 24 h (*n* = 3, unpaired *t*‐test). F) Viability of SH‐SY5Y cells pretreated with 600 nm CsA for 2 h, then treated with 500 µm CuSO_4_ and 150 nm CsA for 24 h (*n* = 3, unpaired *t*‐test). G) ATP levels of SH‐SY5Y cells pretreated 2 h with 1 mm AKB, 100 µm 6‐MP, 400 µm AICAR, and 600 nm CsA, then treated with 250 µm CuSO_4_ for 24 h (n = 3, unpaired *t*‐test). H,I). Western blot analysis (H) and quantification of p‐AMPK/AMPK ratio (I) of SH‐SY5Y cells after 24 h treatment with 1 mm AKB and CuSO_4_. The p‐AMPK/AMPK ratio indicates AMPK activation (*n* = 3, two‐way ANOVA with Tukey's test). Means ± SD. Images of the cell viability assay are shown in the Supplementary Information.

Mechanistically, cytosolic NADH‐reductive stress triggers provoked purine biosynthesis and subsequent ATP depletion, ultimately leading to cell death (**Figure** **4**A).^[^ ^14^, ^15^, ^16^ ^]^ Our study has demonstrated significant cytosolic NADH accumulation during Cu exposure (Figure 3). While multiple studies have documented Cu‐induced ATP depletion and adenosine monophosphate (AMP)‐activated protein kinase (AMPK) activation, our current findings revealed additional metabolic perturbations, including elevated AMP and guanosine monophosphate (GMP) levels in SH‐SY5Y cells following 250 µm CuSO_4_ treatment (Figure 4B).^[^ ^30^, ^31^, ^32^, ^33^ ^]^ In the study of Tsvetkov et al., elesclomol‐Cu pulse treatment induces dramatic upregulation of AMP and depletion of ATP in Cu‐sensitive ABC1 cells but not in Cu‐resistant A549 cells.^[^ ^7^ ^]^ These findings support a NADH‐reductive stress induced by Cu.

To further elucidate the role of NADH‐reductive stress in Cu‐induced cell death, we employed pharmacological interventions targeting distinct aspects of this pathway. AKB, which specifically reduces cytosolic NADH accumulation in our study, significantly attenuated Cu‐mediated cytotoxicity (Figure 4C; Figures S7 and S8, Supporting Information). This protective effect was dose‐dependently reversed by co‐treatment with (R)‐GNE‐140, an effective LDH inhibitor (Figure 4D; Figure S9, Supporting Information), demonstrating that AKB exhibits its cytoprotective effect against Cu through LDH‐dependent NADH conversion.^[^ ^34^ ^]^ Furthermore, the purine biosynthesis inhibitor 6‐mercaptopurine (6‐MP) and the AMPK activator 5‐aminoimidazole‐4‐carboxamide ribonucleoside (AICAR), which attenuates energy stress, similarly protected against Cu‐induced cell death (Figure 4E; Figures S8 and S10, Supporting Information).^[^ ^14^ ^]^ Consistent with our mechanistic hypothesis, CsA, which prevents mitochondrial NADH translocation to the cytosol by inhibiting mPTP opening, also exhibited significant protective effects (Figure 4F; Figures S8 and S11, Supporting Information). Interestingly, both AKB‐mediated NADH reduction and 6‐MP‐induced purine biosynthesis inhibition conferred complete protection against Cu‐induced cytotoxicity during 6 h exposure to 2 mm CuSO_4_ (Figure S12, Supporting Information), suggesting distinct but equally effective mechanisms. To our observation, 6‐MP treatment neither affected Cu‐induced mPTP opening nor altered NADH accumulation (Figure S13, Supporting Information). These findings suggest that the cytoprotective effect of 6‐MP against Cu toxicity operates through a mechanism independent of NADH conversion, likely via its established role in purine biosynthesis inhibition.

Importantly, all 4 compounds (AKB, 6‐MP, AICAR, and CsA) effectively restored Cu‐depleted ATP levels (Figure 4G). We also observed that AKB suppressed Cu‐induced AMPK phosphorylation (Figure 4H,I), indicating that the restoration of cellular ATP levels by AKB administration attenuates the energy stress response triggered by Cu exposure.

The opening of mPTP typically triggers a lethal cellular event, as the leakage of apoptosis‐inducing molecules activates the caspase cascade. However, Cu can suppress apoptosis by inhibiting caspase activity, thereby inducing paraptotic cell death.^[^ ^35^ ^]^ Notably, FMK rescued bortezomib‐induced apoptosis but not Cu‐induced cell death (Figure 1B; Figures S3 and S14, Supporting Information). While the above NADH‐reductive stress‐targeting compounds effectively rescued Cu‐mediated cell death, they demonstrated no comparable protective efficacy against bortezomib‐induced apoptosis (Figure S14, Supporting Information), highlighting fundamental mechanistic differences between Cu‐dependent cell death and classical apoptotic pathways. These findings collectively establish that cuproptosis represents a novel form of regulated cell death mediated through NADH‐reductive stress, involving perturbations in cellular redox homeostasis, purine biosynthesis, and energy metabolism.

## 3 Discussion

Although Cu is an essential trace metal for numerous biological processes, its excessive accumulation can induce neurotoxicity.^[^ ^36^, ^37^ ^]^ The pathological mechanisms of Cu‐mediated neural damage are still poorly understood. Recently, a novel type of regulated cell death, cuproptosis, has been identified. Tsvetkov et al. unexpectedly revealed that this unique form of cell death is mitochondrial respiration‐requiring.^[^ ^7^ ^]^ This finding is particularly intriguing, given that mitochondrial respiration has traditionally been considered a fundamental life‐sustaining process. To elucidate the underlying mechanism of mitochondrial respiration in cuproptosis in neural cells, we used the human neuroblastoma SH‐SY5Y cell line to investigate the involvement of DLD, a key mitochondrial respiratory enzyme, and its metabolic product NADH in Cu pathology.

In our study, Cu was found to mediate mitochondrial DLD activation, thereby inducing cytosolic NADH‐reductive stress, which was closely associated with Cu‐dependent cell death. NADH‐reductive stress‐mediated cuproptosis process involves 3 key mechanistic events: 1) NADH accumulation via Cu‐induced DLD activation in mitochondria, 2) NADH translocation into the cytosol through Cu‐triggered mPTP opening, and 3) NADH‐mediated reductive stress, which promotes purine biosynthesis and energy stress. Notably, pharmacological depletion of cytosolic NADH using AKB rescued cells from Cu‐induced death even in the presence of persistent mPTP opening. Conversely, mPTP inhibitor CsA exhibited protection against Cu toxicity without attenuating Cu‐elevated NADH levels. Additionally, the purine biosynthesis inhibitor 6‐MP attenuated Cu‐induced cell death independently of mPTP opening or NADH accumulation. Collectively, these findings demonstrate that all three events are indispensable for Cu‐induced cell death, and disruption of any single pathway is sufficient to abrogate this regulated cell death mechanism.

While we cannot exclude the possibility that Cu‐induced NADH accumulation may also result from downregulated NADH consumption or confirm that Cu‐activated DLD enhances the TCA cycle. However, we have demonstrated the Cu activated DLD under an alkaline mitochondrial environment, and inhibition of DLD effectively attenuated both Cu‐induced NADH accumulation and subsequent cell death. On the other hand, although other cytotoxic substances may leak into the cytosol through mPTP during Cu exposure, and the traversal of NADH through the mPTP was not directly observed in our study, our findings demonstrate that targeted intervention against NADH‐reductive stress at multiple levels provides robust protection against Cu toxicity. This underlies the central role of NADH‐reductive stress in neural cell cuproptosis. Future studies utilizing advanced techniques such as genetically encoded NADH biosensors targeted to specific subcellular compartments may offer more direct visualization of this process.

Thus, NADH may serve as a potential biological marker for WD. Non‐invasive assessment of cerebral NADH and NAD^+^ levels using^31^ phosphorus magnetic resonance spectroscopy could potentially indicate the likelihood of patients developing neurological and psychiatric symptoms or correlate with disease prognosis.^[^ ^38^ ^]^ Furthermore, targeting NADH‐reductive stress using agents like AKB or 6‐MP, potentially combined with Cu chelators, may offer enhanced neuroprotection during WD treatment and represents a promising avenue for future clinical investigation.

Additionally, our findings demonstrate that cellular sensitivity to Cu is determined not only by Cu transport efficiency within cells (including Cu transporters and chaperones), mitochondrial metabolic factors (including DLD activity and lipoylated enzymes), but also by susceptibility of mPTP to Cu‐induced opening and the cellular capacity to counteract NADH‐reductive stress (including LDH activity, purine biosynthesis, and energy stress response). Our findings highlight the critical role of NADH‐reductive stress in Cu pathology, particularly in modulating neural cell death. However, the generalizability of this specific mechanism to other cell types requires further dedicated investigation. Previous studies have documented cell‐type‐specific variations in intracellular and mitochondrial pH following Cu exposure, which may contribute to differential Cu sensitivity among cell types.^[^ ^23^, ^24^, ^25^ ^]^ Notably, as a critical cellular defense mechanism against NADH‐reductive stress, LDH also plays a crucial role in attenuating Cu‐mediated cell death. Consequently, cells with higher LDH expression may demonstrate enhanced resistance to Cu overload.^[^ ^10^ ^]^ Cu ionophore elesclomol has shown antitumor efficacy in certain cancers and demonstrated preferential therapeutic effects in patients with low LDH levels in a phase III SYMMETRY clinical study.^[^ ^39^, ^40^ ^]^ Mechanistically, LDH inhibition would lead to pyruvate accumulation and entry into the TCA cycle.^[^ ^41^ ^]^ We propose a hypothesis that concomitant administration of LDH inhibitors with elesclomol may increase tumoral sensitivity by redirecting pyruvate flux toward mitochondrial respiration.

We noticed that oligomerized DLAT also exists in cells without high‐dose Cu exposure, and structural predictions from AlphaFill indicate the formation of a disulfide bond between Cys80 and Cys85 in DLD.^[^ ^7^ ^]^ This suggests that Cu‐mediated regulation of DLD activity may represent a physiological mechanism. Notably, we observed that 15 µm CuSO_4_ treatment increased cell viability by ≈20%, implying that transient, moderate cellular Cu accumulation might enhance mitochondrial respiration without inducing mPTP opening, thereby promoting cellular metabolic activity. However, when cellular Cu accumulation reaches a threshold sufficient to trigger mPTP opening, the consequent NADH translocation induces pathological reductive stress. The elucidation of this NADH‐dependent regulatory mechanism provides new insights into both physiological and pathological aspects of cellular Cu homeostasis, opening avenues for further investigation into the dual roles of Cu in cellular metabolism.

## 4 Conclusion

**Scheme 1:**
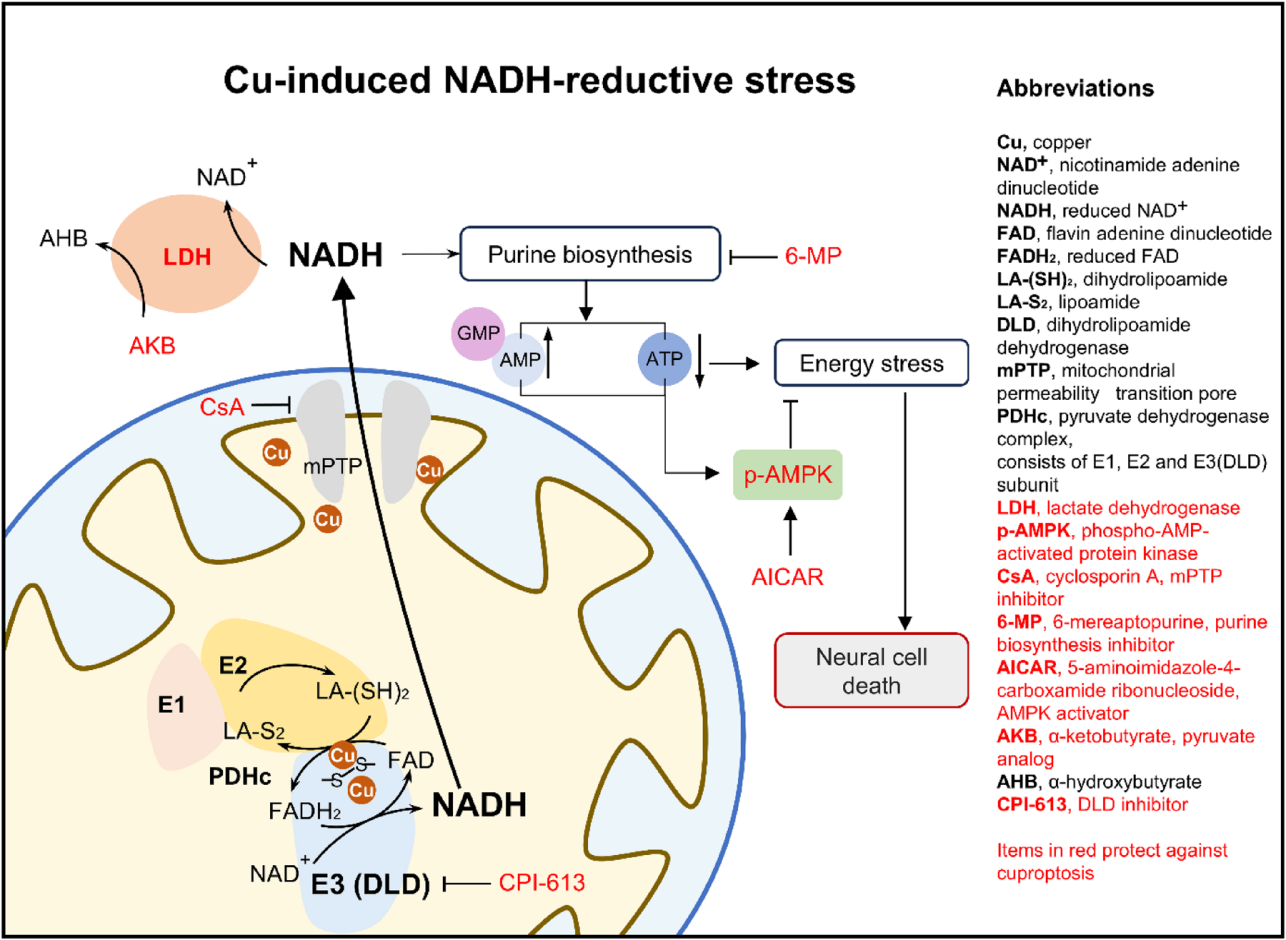
Schematic of the molecular mechanism of Cu‐induced NADH‐reductive stress leading to cuproptosis in neural cells. Mitochondrial Cu accumulation activates DLD through disulfate bond formation and promotes NADH generation. The excess NADH subsequently translocates to the cytosol following Cu‐induced mPTP opening, which drives NADH‐reductive stress. The cytosol NADH accumulation provokes the purine biosynthesis pathway and triggers massive ATP consumption, ultimately leading to neural cell death. This Cu‐NADH‐reductive stress‐mediated cell death can be suppressed by inhibition of DLD or mPTP, deletion of cytosolic NADH, disruption of purine biosynthesis, and activation of AMPK.

In summary, the current study elucidates the underlying mechanism by which DLD regulates cuproptosis in human neuroblastoma SH‐SY5Y cells. We demonstrate that Cu induces DLD activation through disulfate bond formation under alkaline mitochondrial pH conditions. The resultantly accumulated NADH subsequently translocates to the cytosol via Cu‐mediated mPTP opening, thereby driving NADH‐reductive stress (**Scheme** **1**). Our findings highlight the critical role of NADH‐reductive stress in Cu pathology, particularly in modulating cell death. These insights may provide novel therapeutic strategies for WD and enhance the efficacy of Cu ionophore‐based cancer therapies.

## 5 Experimental Section

### Materials and Reagents

Copper sulfate pentahydrate (C7850), dipotassium hydrogen phosphate (D8490), potassium dihydrogen phosphate (P7392), EDTA disodium salt (E8030), NAD^+^ (N8110), and sodium pyruvate (P8380) were purchased from Solarbio. 0.5 m DTT (ST041) was purchased from Beyotime. α‐ketobutyrate (K401) and dihydrolipoic acid (437694) were purchased from Sigma. N‐acetylcysteine (HY‐B0215), 6‐mercaptopurine (HY‐13677), ammonium tetrathiomolybdate (HY‐W076067), BOC‐D‐FMK (HY‐13229), ferrostatin‐1 (HY‐100579), CPI‐613 (HY‐15453), cyclosporin A (HY‐B0579), (R)‐GNE‐140 (HY‐100742A), AICAR (HY‐13417), and bortezomib (HY‐10227) were purchased from Med Chem Express.

### Cell Culture and Treatment

SH‐SY5Y cell line (RRID:CVCL_0019) was obtained from the Cell Bank of the Chinese Academy of Sciences (SCSP‐5014). Cells were maintained in a growing medium consisting of Dulbecco's modified Eagle's medium/Ham's F 12 nutrient medium (DMEM/F12) (Procell, PM150312) with 15% fetal bovine serum (Vivacell, C04001) and 1% non‐essential amino acids (Biosharp, BL1115A) at 37 °C within a humidified atmosphere of 20% O_2_ and 5% CO_2_. Since pyruvate affects the accumulation of NADH and cell viability under Cu treatment (Figure S15, Supporting Information), the DMEM/F12 growing medium was replaced with pyruvate‐free DMEM (Vivacell, C3110) during the treatment to avoid the interference of pyruvate.^[^ ^14^ ^]^

CuSO_4_ resolved in ddH_2_O was used to achieve a Cu exposure environment. Elesclomol‐Cu treatment was carried out with 50 nm elesclomol and 50 µm CuSO_4_. For pulse treatment, the treatment medium was discarded after treatment for 2 h, and cells were maintained in pyruvate‐free DMEM for another 22 h before harvesting. The cell death modulating compounds, including Fer‐1, FMK, NAC, and Cu chelator TTM were pretreated for 2 h following co‐treatment with CuSO_4_ or elesclomol‐Cu. The mPTP inhibitor CsA needed pretreatment with a dose of 600 nm for 2 h, and the following treatment dose was changed to 150 nm with or without CuSO_4_ and elesclomol.

### Cell Viability Assay

Cell viability was measured using Calcein AM/PI cell viability assay kit (Beyotime, C2015M) together with Hoechst 33342 (Beyotime, C1022) staining. Briefly, cells were seeded into a 96‐well culture plate with a density of 1.5  ×  10^4^ cells per well. After treatment, cells were incubated in staining solution containing Calcein‐AM (1∶2000), PI (1∶1000), and Hoechst 33342 (5 µg ml^−1^) at 37 °C for 30 min. Finally, images of the live (green fluorescence), dead (red fluorescence) cells and nuclei (blue) were obtained using an Echo Revolve Hybrid Microscope and are shown in Supplementary Information. The cell viability was quantitatively analyzed by ImageJ software (National Institutes of Health).

### Western Blotting and Antibodies

Cells were seeded into a 6‐well culture plate with a density of 1  ×  10^6^ cells per well. After treatment, cells were washed with PBS, then lysed using RIPA (Beyotime, P0013B) lysis buffer containing protease and phosphatase inhibitor cocktail (Beyotime, P1045). The protein concentration was determined using the super‐rapid protein quantification kit (BCA) (Abbkine, KTD3010) with a Tecan infinite F50 microplate reader (Tecan). Samples were loaded into Bis‐tris PAGE gels and then transferred to 0.22 µm PVDF membranes (Millipore, ISEQ00010). Membranes were blocked for 1 h in 5% BSA solution at room temperature and then incubated in the indicated primary antibodies at 4 °C overnight. After washing, the membranes were incubated with secondary antibodies for 1 h at room temperature before washing and visualization with Tanon Femto‐sig ECL (Tanon, 180–506) using Tanon 5200 automatic multifunctional imaging system (Tanon). DLAT (YM1328, 1∶1000), FDX1 (YT8131, 1∶500), LIAS (YT8133, 1∶2000), DLD (YT8134, 1∶500), AMPK α1/2 (Phospho‐Thr183/Thr172) (YM8689, 1:4000), AMPKα1/2 (YT0216, 1∶1000) were obtained from Immunoway. β‐actin (66009‐1‐Ig, 1∶5000) was obtained from PTG. The goat anti‐rabbit IgG/HRP (A21020, 1∶10000) and goat anti‐mouse IgG/HRP (A21010, 1∶10000) secondary antibodies were obtained from Abbkine. The blots were quantitatively analyzed by ImageJ software (National Institutes of Health), and the protein amounts were normalized to β‐actin.

### NADH/NAD^+^ Measurement

Cellular NADH levels and NADH/NAD^+^ ratios were measured using the NAD^+^/NADH assay kit with WST‐8 (Beyotime, S0175) according to the manufacturer's instructions with modifications. The entire extraction process should be protected from light. Briefly, 3 × 10^5^ cells were seeded in a 24‐well plate. After an 18 h treatment, the cells were quickly washed with PBS, and then ice‐cold lysis buffer was added to lyse the cells. After 10 min centrifugation at 10 000 × g under 4 °C, part of the cell lysate supernatant was transferred to an empty well of 96‐well plates for 30 min incubation at 75 °C to dismiss NAD^+^. The supernatant with or without 75 °C incubation was used to determine the total NAD and NADH, respectively. Then, ethanol dehydrogenase was added to convert NAD^+^ from total NAD into NADH, which was detected with WST‐8. The amount of total NAD and NADH was determined by measurement of absorbance at 450 nm, normalized by protein concentration acquired by BCA assay. A standard curve of NADH was generated following the manufacturer's instructions.

Equation:

(1) [NAD+]=[NADtotal]−[NADH]

(2) [NADH]/[NAD+]=[NADH]/([NADtotal]−[NADH])

### DLD Activity Assay

The DLD activity assay method primarily references the research of Patel et al., with modifications.^[^ ^19^ ^]^ The SH‐SY5Y cells were collected and washed three times with PBS, then resuspended in lysis buffer containing 10 mm potassium phosphate (pH *8.0*), 150 µm EDTA, and protease inhibitors, followed by sonication. The lysate was then centrifuged, and the supernatant was the DLD extracts used for DLD activity measurement. The protein concentrations of the extracts were determined using the BCA assay before measuring DLD activity. The DLD activity assay buffer consists of 0.1 m potassium phosphate (pH *8.0*), 1.5 mm EDTA, 12 mm DHLA, 3 mm NAD^+^, and 0.6 µg total protein from CuSO_4_‐treated cells. The reaction mixture was prepared on ice, avoiding the light. And the reaction was initiated by adding the extract, followed by incubation at 37 °C. The absorbance at 340 nm after 30 min incubation was measured using a micro‐UV spectrophotometer. The DLD kinetics were performed in assay buffer (pH *8.0* or *7.2*) with various concentrations of DHLA at 37 °C for 1 min. The *K_M_* and *V_max_* were obtained by fitting the Michaelis–Menten curve. For enzyme kinetics after DTT reduction of disulfate bonds, the DLD extracts were incubated with or without 0.1 m DTT for 1 h after an overnight 15 µm CuSO_4_ or ddH_2_O incubation at 37 °C, and then implied for kinetic assays in assay buffer (pH *8.0*).

### Mitochondria Isolation

The mitochondria isolation was conducted by using ExKine mitochondrion extraction kit for cultured cells (Abbkine, KTP4003) according to the manufacturer's instructions. Briefly, 1 × 10^6^ cells were seeded in a 6‐well plate. After treatment, cells were harvested and washed by suspending the cell pellet in ice‐cold PBS, followed by centrifugation at 1000  ×  g for 3 min at 4 °C, after which the PBS was discarded. The cell pellets are resuspended in cold lysis buffers A, B, and C, and the mixture is centrifuged at 600  ×  g for 10 min at 4 °C. Finally, the supernatant was collected and centrifuged at 3000  ×  g for 15 min at 4 °C for higher purity. The isolated mitochondria in pellets were resuspended in storage buffer for the following BCECF staining.

### The BCECF Staining

The cellular and mitochondrial pH were measured using a pH‐sensitive fluorescent probe BCECF‐AM (Beyotime, S1006).^[^ ^42^ ^]^ The isolated mitochondria were incubated with mitochondria storage buffer containing BCECF AM (2.5 µm) and Mito‐Tracker Red CMXRos (100 nm, Beyotime, C1035) that probe active mitochondria for 30 min in the cell incubator. The suspension was then centrifuged at 9000  ×  g for 5 min to remove excess probe. Mitochondria were then resuspended in storage buffer, and the BCECF fluorescence (green), which increases with increasing pH, and Mito‐Tracker Red fluorescence (red) were measured with an Echo Revolve Hybrid Microscope.

The cellular pH was measured in the living cells. The cells were seeded into a 96‐well plate with a density of 1 × 10^4^ cells/well. After treatment, cells were probed with DMEM containing BCECF AM (2.5 µm) and Mito‐Tracker Red CMXRos (100 nm). After three washes with PBS, the BCECF fluorescence (green) and Mito‐Tracker Red fluorescence (red) were measured with an Echo Revolve Hybrid Microscope.

The pH of CuSO_4_‐treated DMEM medium was measured after 30 min incubation with BCECF AM (2.5 µm) using an Echo Revolve Hybrid Microscope.

The mean fluorescence intensity was quantitatively analyzed by ImageJ software (National Institutes of Health).

### mPTP State Measurement

The mPTP opening of SH‐SY5Y cells was detected using the mPTP assay kit (Beyotime, C2009S) according to the manufacturer's instructions with modifications. Briefly, 1 × 10^4^ cells were seeded into a 96‐well plate. Then, cells were treated with or without 0.6 µm CsA for 2 h. Then the media were changed with 15, 250 µm CuSO_4,_ 150 nm CsA or 1 mm AKB for 24 h treatment. After treatment, the cells were incubated with staining solution containing Calcein AM (1∶10000) and Mito‐Tracker Red CMXRos (100 nm), incubated at 37 °C for 10 min, followed by the replacement of the staining solution with quenching solution containing CoCl_2_ (1∶100) and 15, 250 µm CuSO_4,_ 150 nm CsA or 1 mm AKB in DMEM for another 30 min incubation at 37 °C. Cells were then washed three times with DMEM, and the Calcein fluorescence (green) and Mito‐Tracker Red fluorescence (red) were observed under an Echo Revolve Hybrid Microscope.

### AMP and GMP Measurement

The AMP and GMP levels were measured using LC/MS analysis performed on a 5500 QTRAP mass spectrometer (AB SCIEX) coupled to an Agilent 1290 Infinity LC UPLC system (Agilent). Cells were grown in 100 mm dishes at a density of 5 × 10^6^ cells/dish. 24 h after treatment with or without 250 µm CuSO_4_, cells were washed 3 times with chilled PBS, and metabolites were extracted with 1 mL of methanol‐acetonitrile solution (1:1, v/v, prechilled to – 80 °C). After extraction, supernatants were nitrogen‐dried and stored at ‐80 °C. Dried polar samples were resuspended in 100 µL methanol‐acetonitrile solution (1:1, v/v), and 2 µL was injected into an ACQUITY UPLC BEH Amide 1.7 µm, 2.1 mm × 100 mm column for separation with column temperature at 45 °C. Mobile phase: A was a 10 mm ammonium acetate aqueous solution, B was acetonitrile. The gradient program was as follows: 0–18 min, B decreased linearly from 90% to 40%; 18–18.1 min, B increased linearly from 40% to 90%; 18.1–23 min, B remained at 90%. A standard mixture was included for chromatographic retention time correction. Mass spectrometric analysis was performed using the 5500 QTRAP mass spectrometer (AB SCIEX) in negative ion mode. ESI source conditions were as follows: source temperature 450 °C, Ion Source Gas1 (Gas1): 45, Ion Source Gas2 (Gas2): 45, Curtain gas (CUR): 30, Ion Spray Voltage Floating (ISVF): ‐4500 V. Multiple Reaction Monitoring (MRM) mode was used to detect target ion pairs. Retention times and ion transitions of all metabolites were validated according to chemical standards.

### ATP Measurement

The intracellular ATP level was measured using an ATP colorimetric assay kit (Elabscience, E‐BC‐K157‐S) according to the manufacturer's instructions. Briefly, cells were seeded into a 6‐well plate with a density of 1  ×  10^6^ cells per well. The cells were washed and collected after 24 h treatment, then lysed using cell lysis buffer to release ATP. The cell lysate was centrifuged at 10000 × g for 5 min at 4 °C, and the supernatant was then used for ATP measurement following the manufacturer's instructions. The absorbance at 636 nm was measured using a micro‐UV spectrophotometer. The obtained ATP level was normalized with the protein concentration acquired by the BCA assay.

### Molecular Docking

The interaction of DLD between Cu ion and its cofactors was analyzed using the AlphaFill databank (https://alphafill.eu), which analyzes protein‐ligand interactions based on predicted protein models in the AlphaFold protein structure database. The model of the DLD protein was retrieved using the AlphaFold identifier (Uniprot ID: P09622). The prediction of potential DLD binding sites was conducted with a 25% identity threshold that is close to the minimal sequence identity requirement for structural homology.^[^ ^26^ ^]^ The results of predicted Cu‐DLD alignment (PDB ID: 6KYY.B, Asym: AV) and alignment involved FAD (PDB ID: 3RNM.A, Asym: B) and NAD (PDB ID: 2EQ7.A, Asym: N) were visualized using AlphaFill.

### Statistical Analysis

Plots and statistical analysis were performed with Graph‐Pad Prism 9.0 (Graph Pad), and the results were presented as the Mean ± SD. Statistical significance was calculated as appropriate using an unpaired two‐tailed *t*‐test, one‐way ANOVA with Dunnett's test, or two‐way ANOVA with Tukey's test as indicated in the figure legends. The value of *p* <0.05 was considered significant.

## Conflict of Interest

The authors declare no conflict of interest.

## Supporting information



Supporting Information

Supporting Information

## Data Availability

The data that support the findings of this study are available from the corresponding author upon reasonable request.
