# Supplementary material for: NADH‐Reductive Stress Induced by Dihydrolipoamide Dehydrogenase Activation Contributes to Cuproptosis
Source: Adv Sci (Weinh). 2025 Dec 5;13(9):e20444. doi: 10.1002/advs.202520444 (PMC12904017; doi:10.1002/advs.202520444)
Supplement: Supplementary file 1 — Supporting Information [file ADVS-13-e20444-s002.docx]

Supporting Information

NADH-reductive stress induced by dihydrolipoamide dehydrogenase activation contributes to cuproptosis

*Si-Yi Zhang, Xing-Hua Ren, Cheng-Hong Zhang, Zhan-You Wang**

Contents:

Page 2: **Figure S1.** Images of cell viability in Figure 1A.

Page 3: **Figure S2.** Images of cell viability in Figure 1B.

Page 4: **Figure S3.** Viability of cells treated with TTM, Fer-1, FMK, NAC and elesclomol- Cu.

Page 5: **Figure S4.** Images of cell viability in Figure 1F.

Page 6: **Figure S5.** Celluar and medium pH after CuSO_4_ treatment.

Page 7: **Figure S6.** The mPTP state of cells treated with 15 μM CuSO_4_.

Page 8: **Figure S7.** Images of cell viability in Figure 4C.

Page 9: **Figure S8.** Viability of cells treated with AKB, 6-MP, AICAR, CsA and elesclomol-Cu.

Page 10: **Figure S9.** Images of cell viability in Figure 4D.

Page 11: **Figure S10.** Images of cell viability in Figure 4E.

Page 12: **Figure S11.** Images of cell viability in Figure 4F.

Page 13: **Figure S12.** Viability of cells treated 6 h with AKB, 6-MP, AICAR and 2 mM CuSO_4_.

Page 14: **Figure S13.** The NADH level and mPTP state of SH-SY5Y cells treated with 6-MP and 250 μM CuSO_4_.

Page 15: **Figure S14.** Viability of cells treated with AKB, 6-MP, AICAR, CsA, Fer-1, FMK and bortezomib.

Page 16: **Figure S15.** Viability of cells treated with sodium pyruvate and 2 mM CuSO_4_.


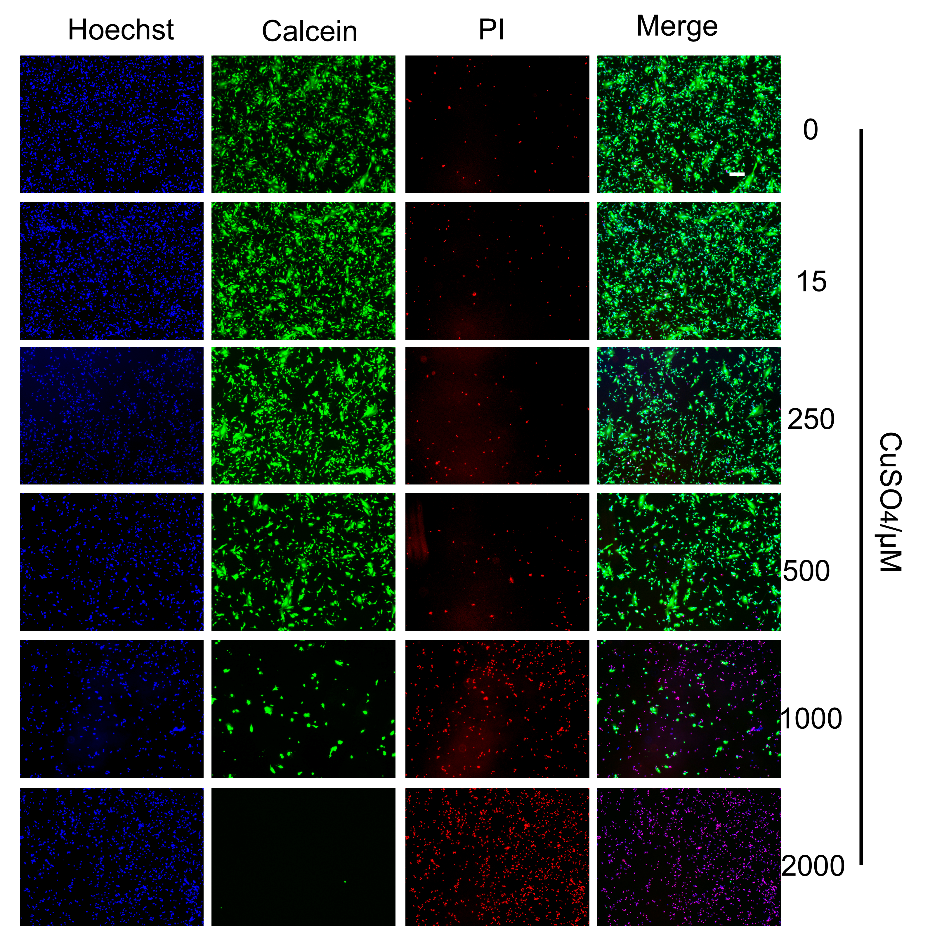


**Figure S1.** Images of cell viability in Figure 1A. Calcein fluorescence (green), PI (red), Hoechst 33342(blue). Scale bar, 100 μm.


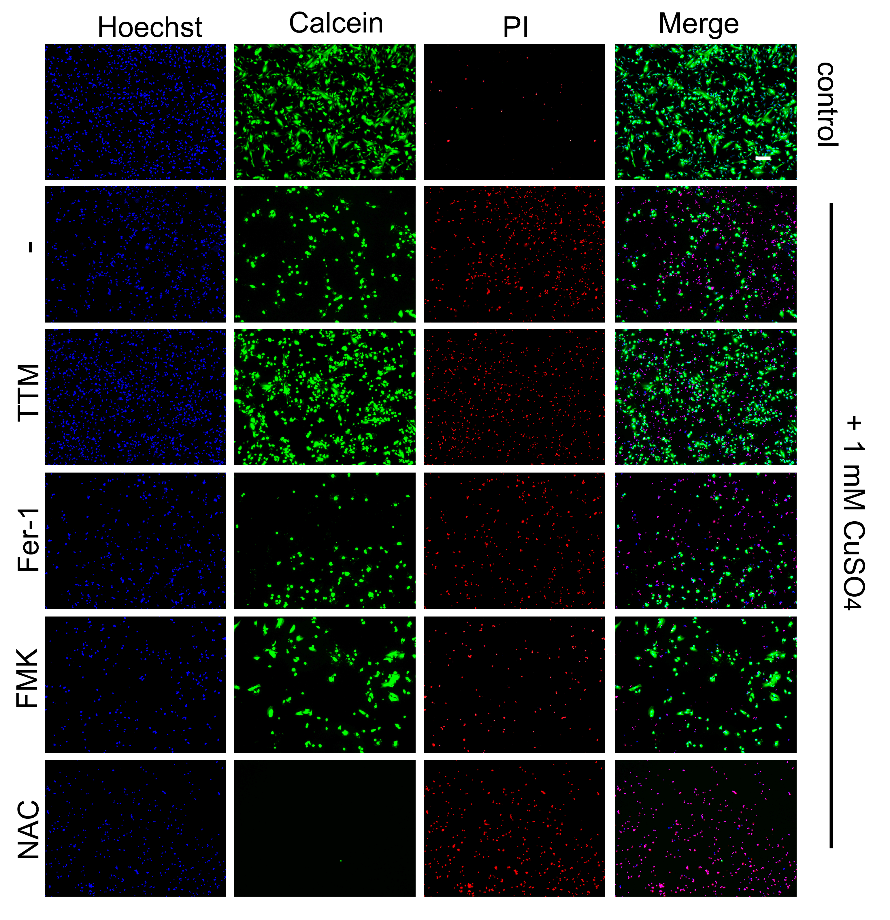


**Figure S2.** Images of cell viability in Figure 1B. Calcein fluorescence (green), PI (red), Hoechst 33342(blue). Scale bar, 100 μm.


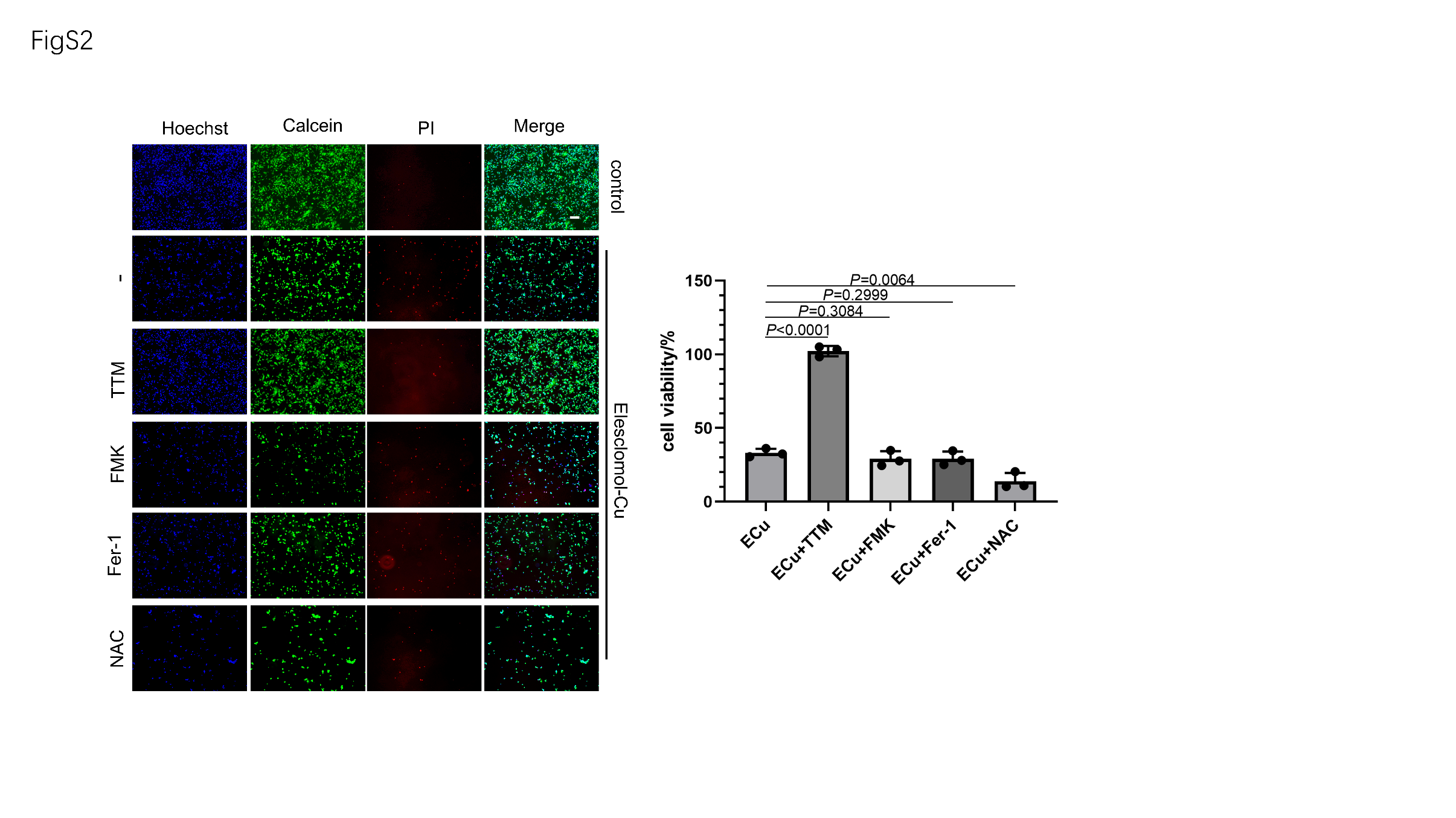


**Figure S3.** Viability of cells pretreated 2 h with 5 μg/ml TTM, 10 μM Fer-1, 50 μM FMK, 5 mM NAC, then treated with 50 nM elesclomol- 50 μM CuSO_4_ (ECu) for 24 h (unpaired *t*-test). Means ± SD. n=3. Calcein fluorescence (green), PI (red), Hoechst 33342(blue). Scale bar, 100 μm.


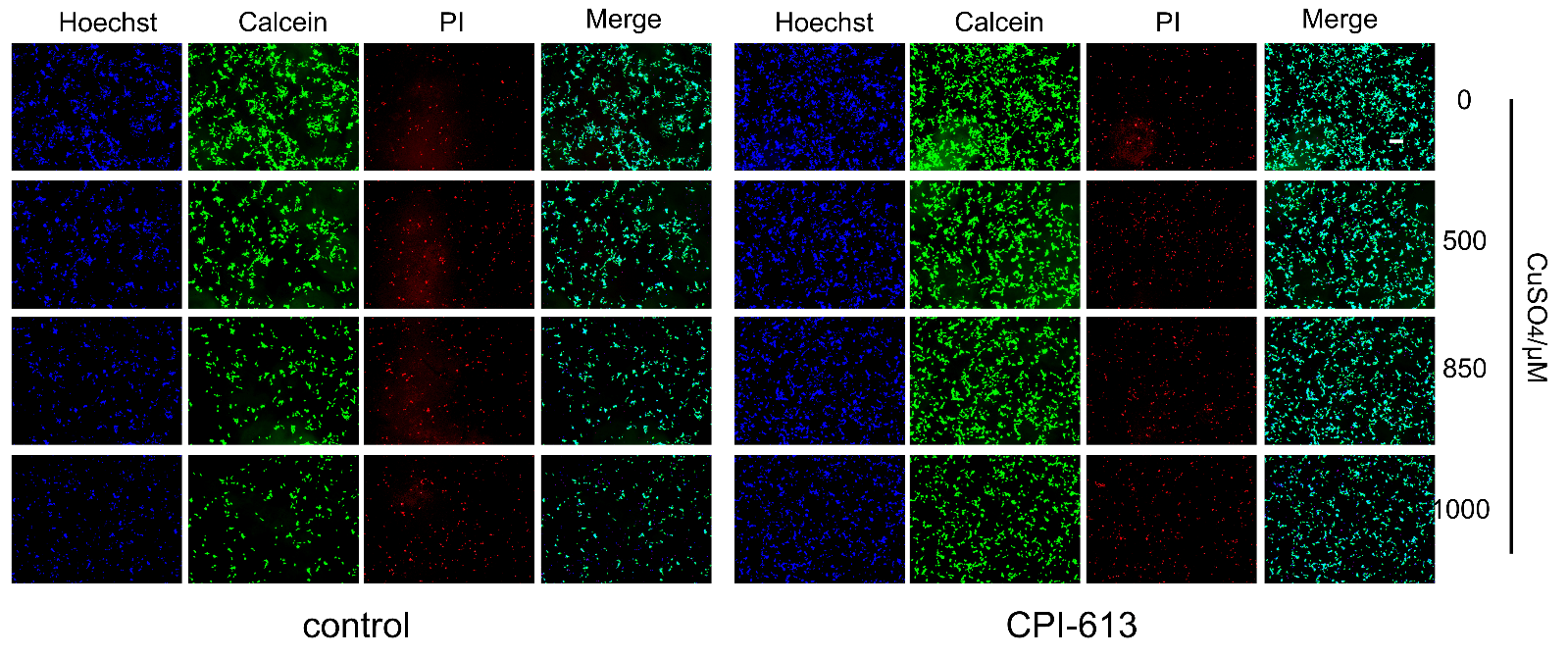
 **Figure S4.** Images of cell viability in Figure 1F. Calcein fluorescence (green), PI (red), Hoechst 33342(blue). Scale bar, 100 μm.


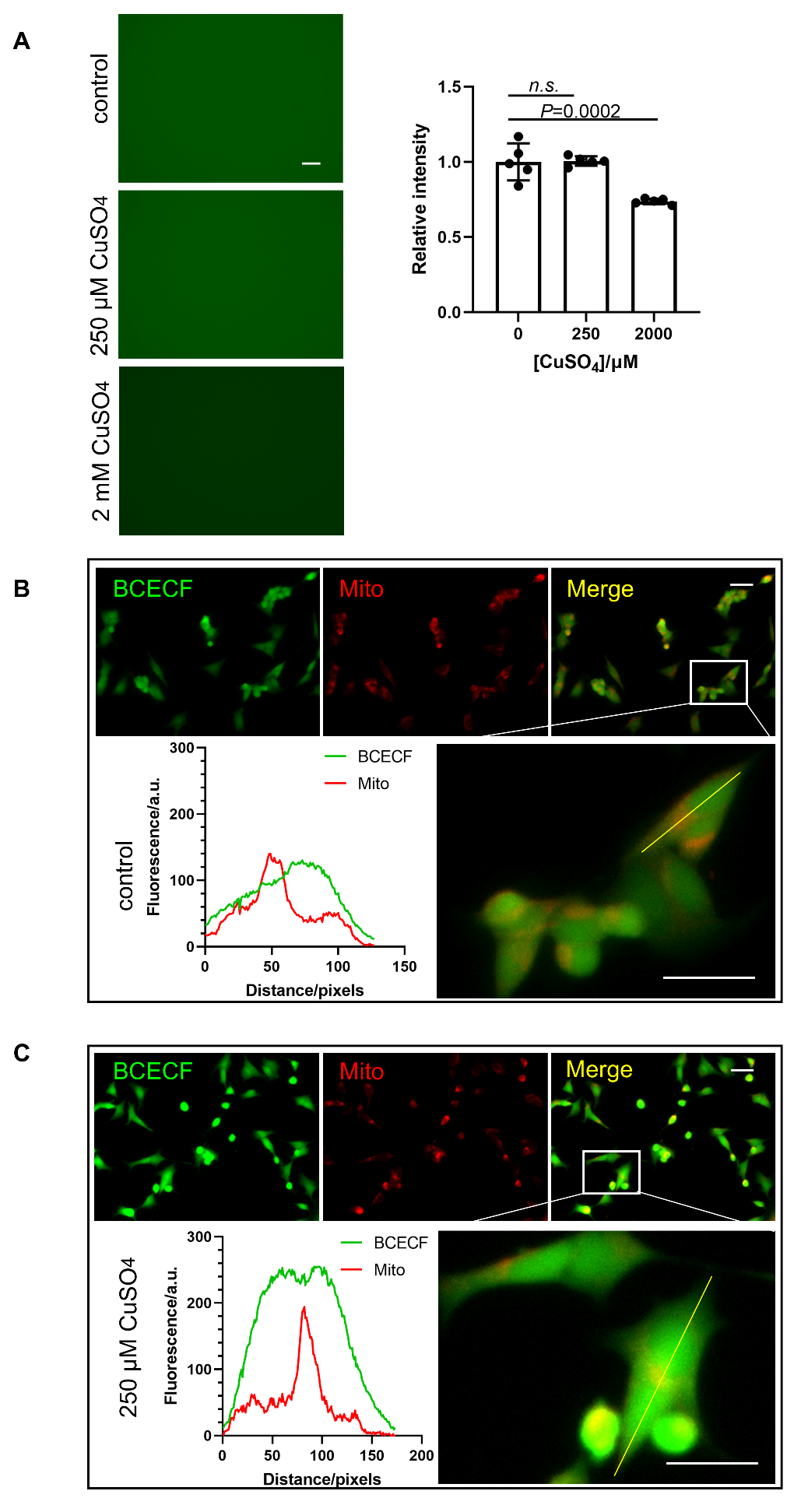


**Figure S5.** Celluar and medium pH after CuSO_4_ treatment. (A) The pH of DMEM medium after 24-hour CuSO_4_ treatment. BCECF-AM (green). Means ± SD. n=5.One-way ANOVA. (B-C). Celluar pH after 24-hour CuSO_4_ treatment. The plots showed the signal distribution of the yellow line in the images. Control (B), 250μM CuSO_4_ (C). BCECF-AM (green), Mito-Tracker Red CMXRos (Mito, red). Scale bar, 50 μm.


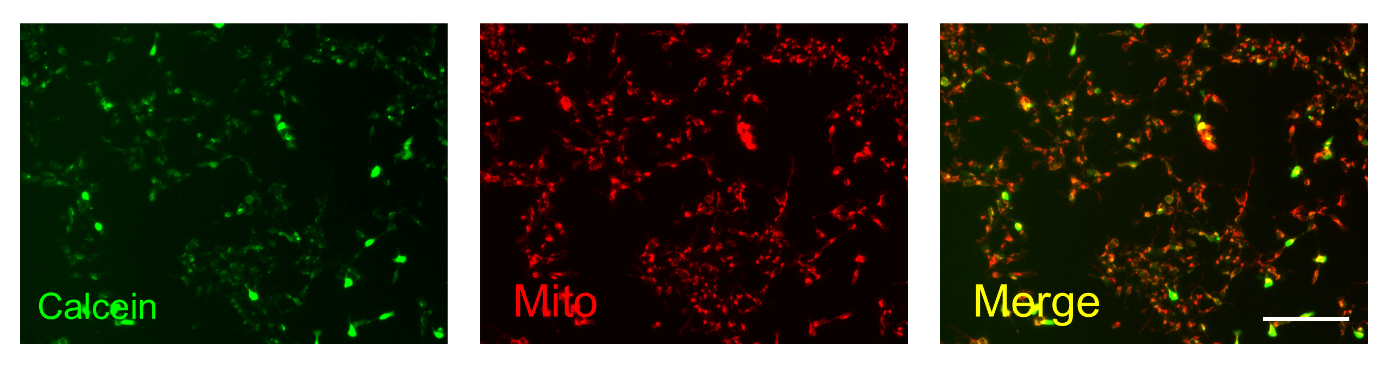


**Figure S6.** The mPTP state of cells treated 24 h in 15 μM CuSO_4_. Calcein fluorescence (green), Mito-Tracker Red CMXRos (Mito, red). Scale bar, 100 μm.


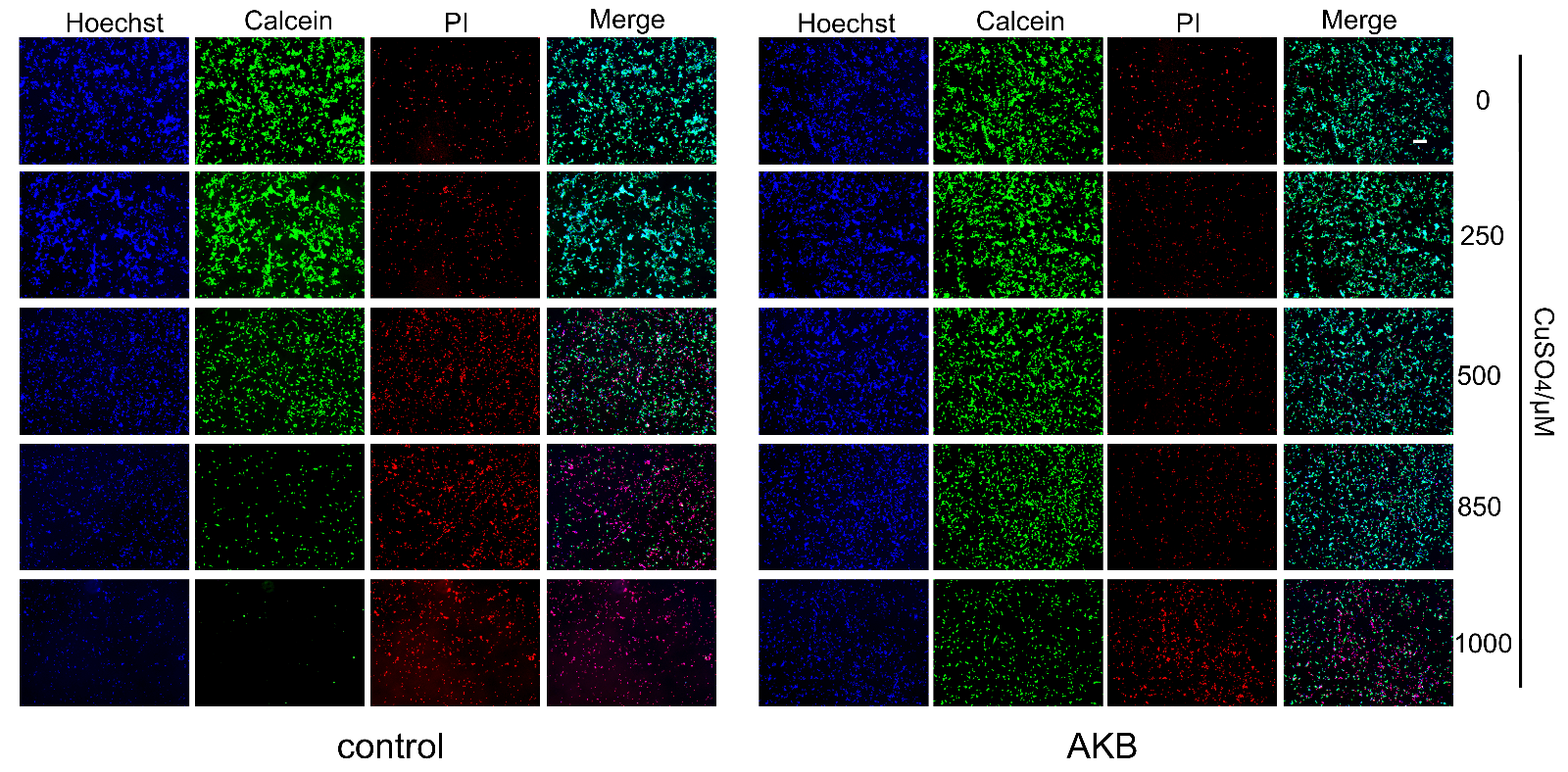


**Figure S7.** Images of cell viability in Figure 4C. Calcein fluorescence (green), PI (red), Hoechst 33342(blue). Scale bar, 100 μm.


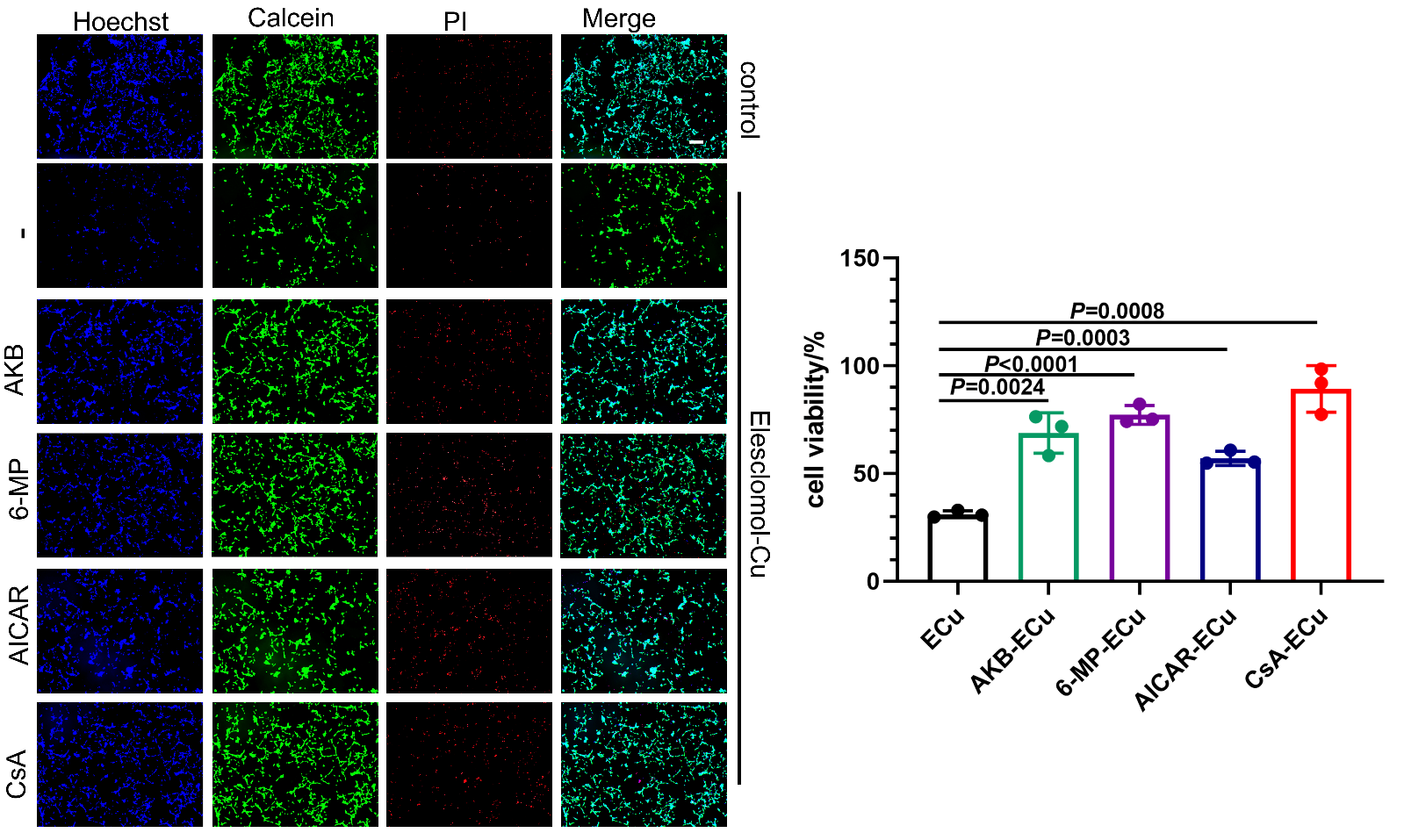


**Figure S8.** Viability of cells pretreated 2 h with 1 mM AKB, 100 μM 6-MP, 400 μM AICAR and 600 nM CsA, then treated with 50 nM elesclomol - 50 μM CuSO_4_ (ECu) for 24 h (unpaired *t*-test). Means ± SD. n=3. Calcein fluorescence (green), PI (red), Hoechst 33342(blue). Scale bar, 100 μm.


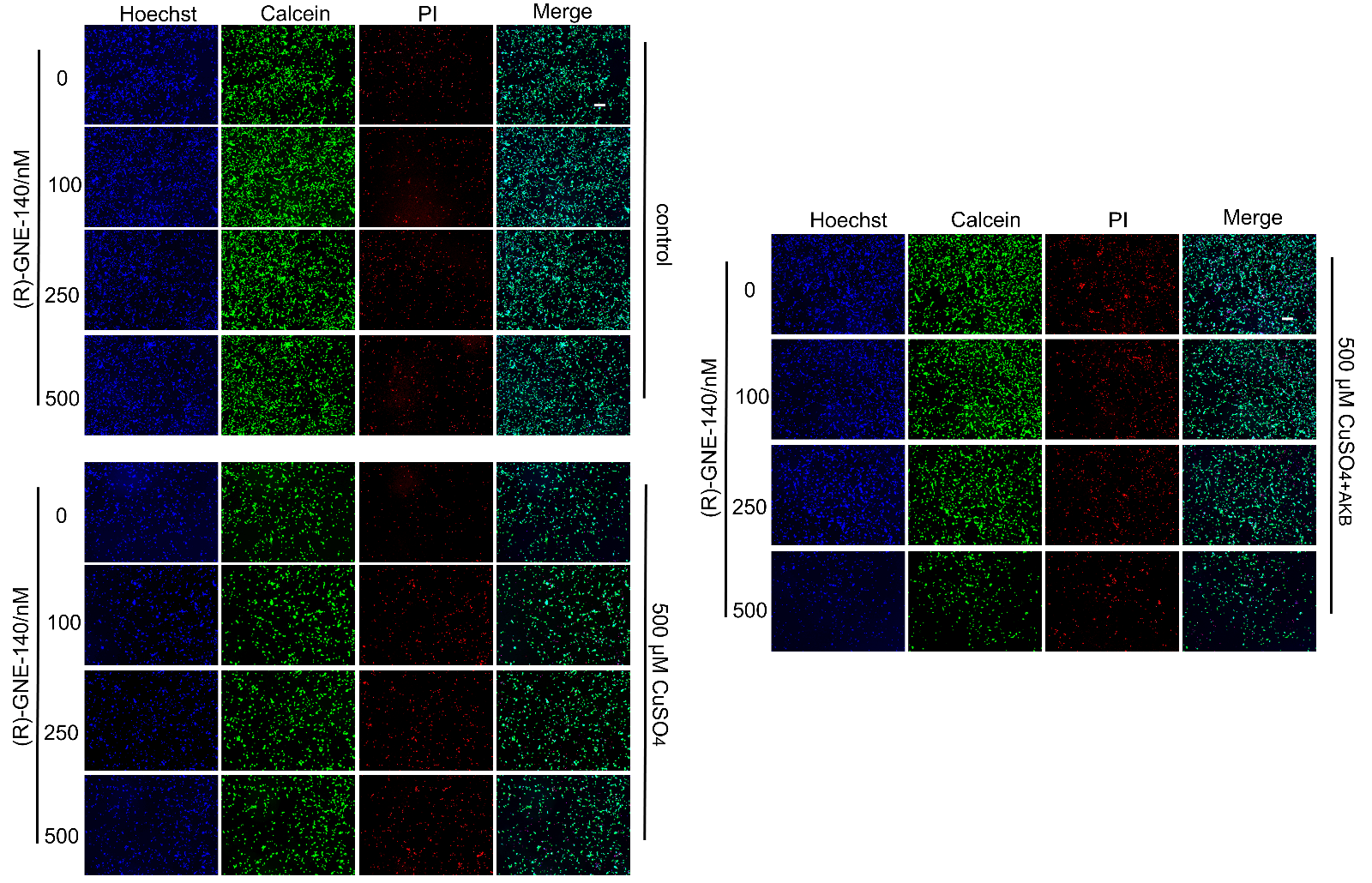


**Figure S9.** Images of cell viability in Figure 4D. Calcein fluorescence (green), PI (red), Hoechst 33342(blue). Scale bar, 100 μm.


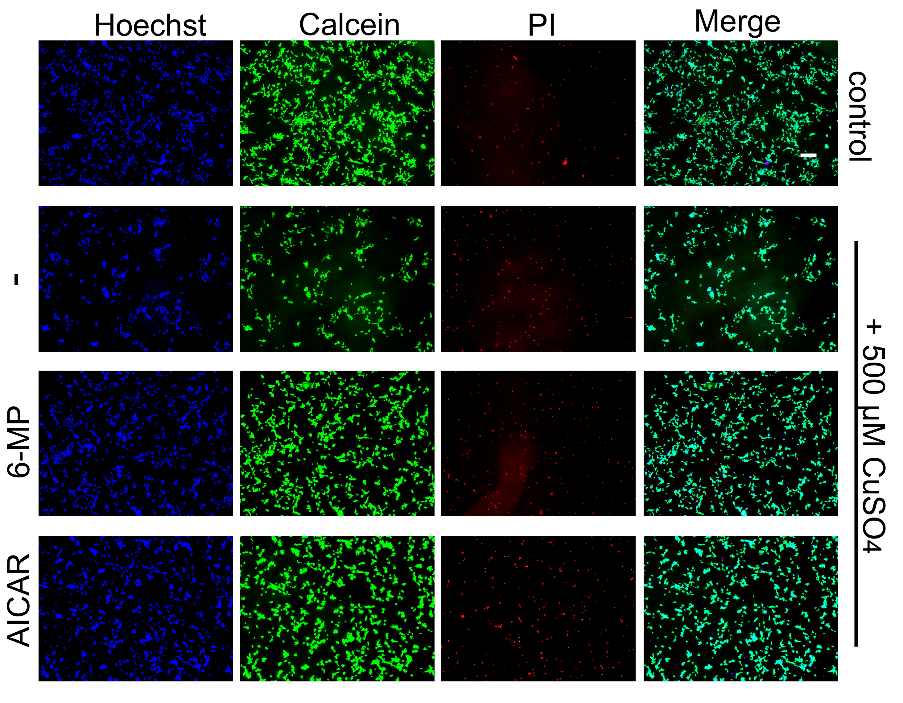


**Figure S10.** Images of cell viability in Figure 4E. Calcein fluorescence (green), PI (red), Hoechst 33342(blue). Scale bar, 100 μm.


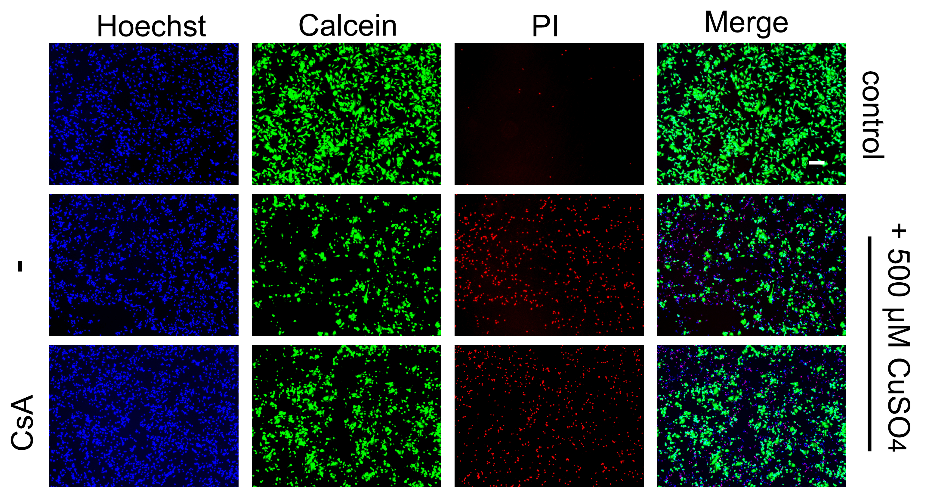


**Figure S11.** Images of cell viability in Figure 4F. Calcein fluorescence (green), PI (red), Hoechst 33342(blue). Scale bar, 100 μm.


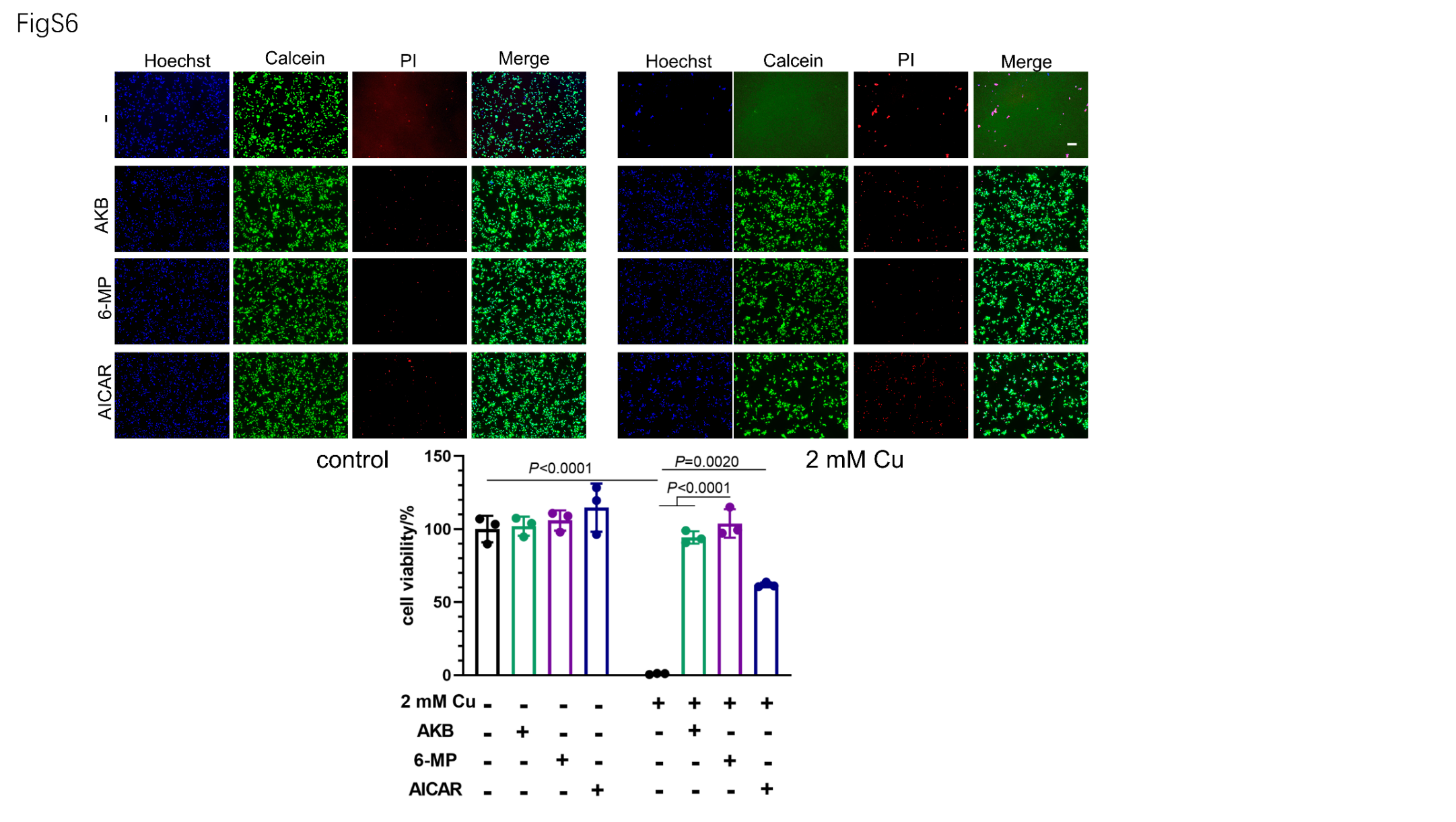


**Figure S12.** Viability of cells treated 6 h with 1 mM AKB, 100 μM 6-MP, 400 μM AICAR and 2 mM CuSO_4_ (unpaired *t*-test). Means ± SD. n=3. Calcein fluorescence (green), PI (red), Hoechst 33342(blue). Scale bar, 100 μm.


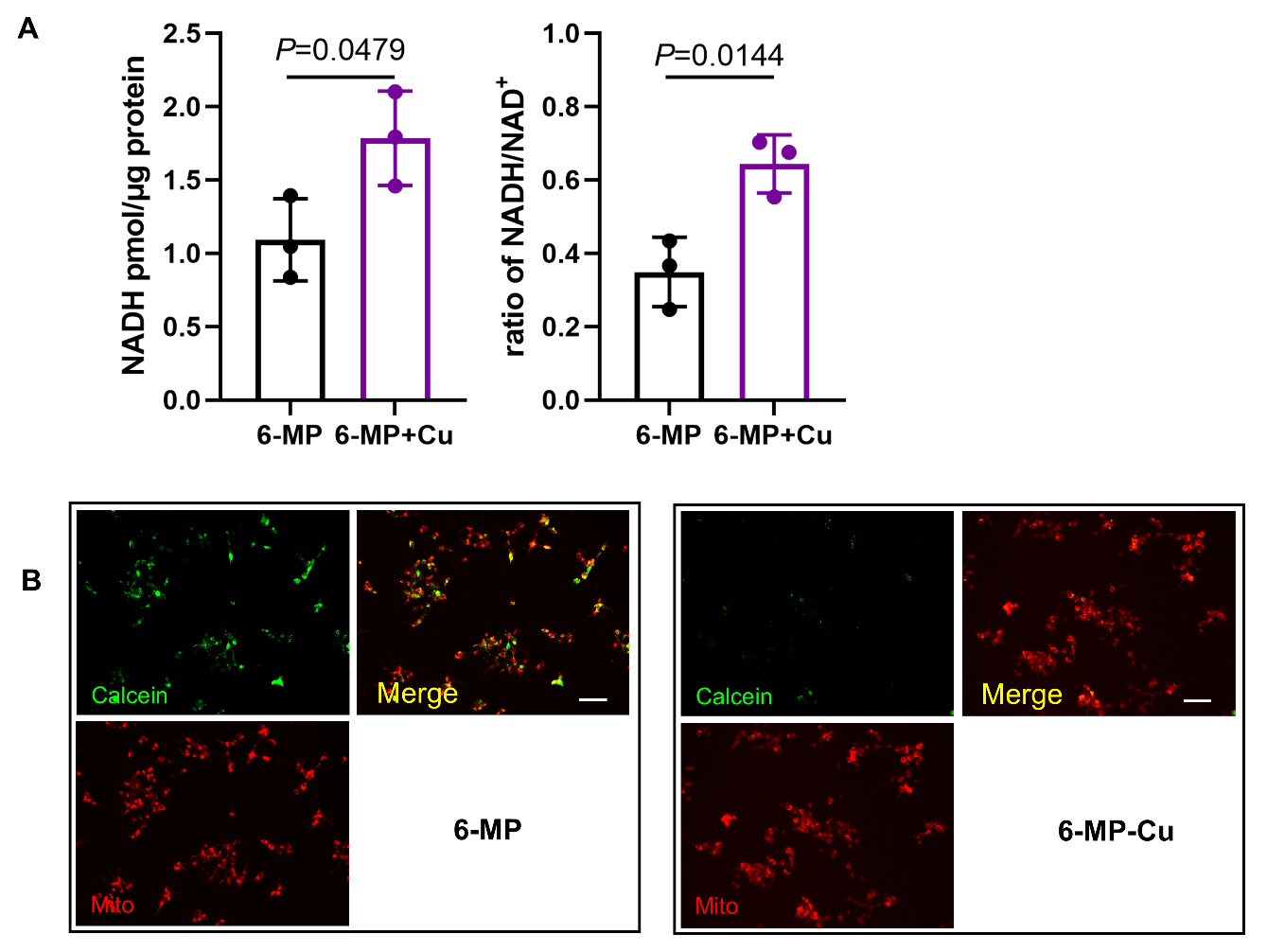


**Figure S13.** The NADH level and mPTP state of SH-SY5Y cells treated 24 h with 100 μM 6-MP and 250 μM CuSO_4_. (A) The NADH level and NADH/NAD^+^ ratio (unpaired *t*-test).Means ± SD. n=3.(B) The mPTP state of cells. Calcein fluorescence (green), Mito-Tracker Red CMXRos (Mito, red). Scale bar, 100 μm.


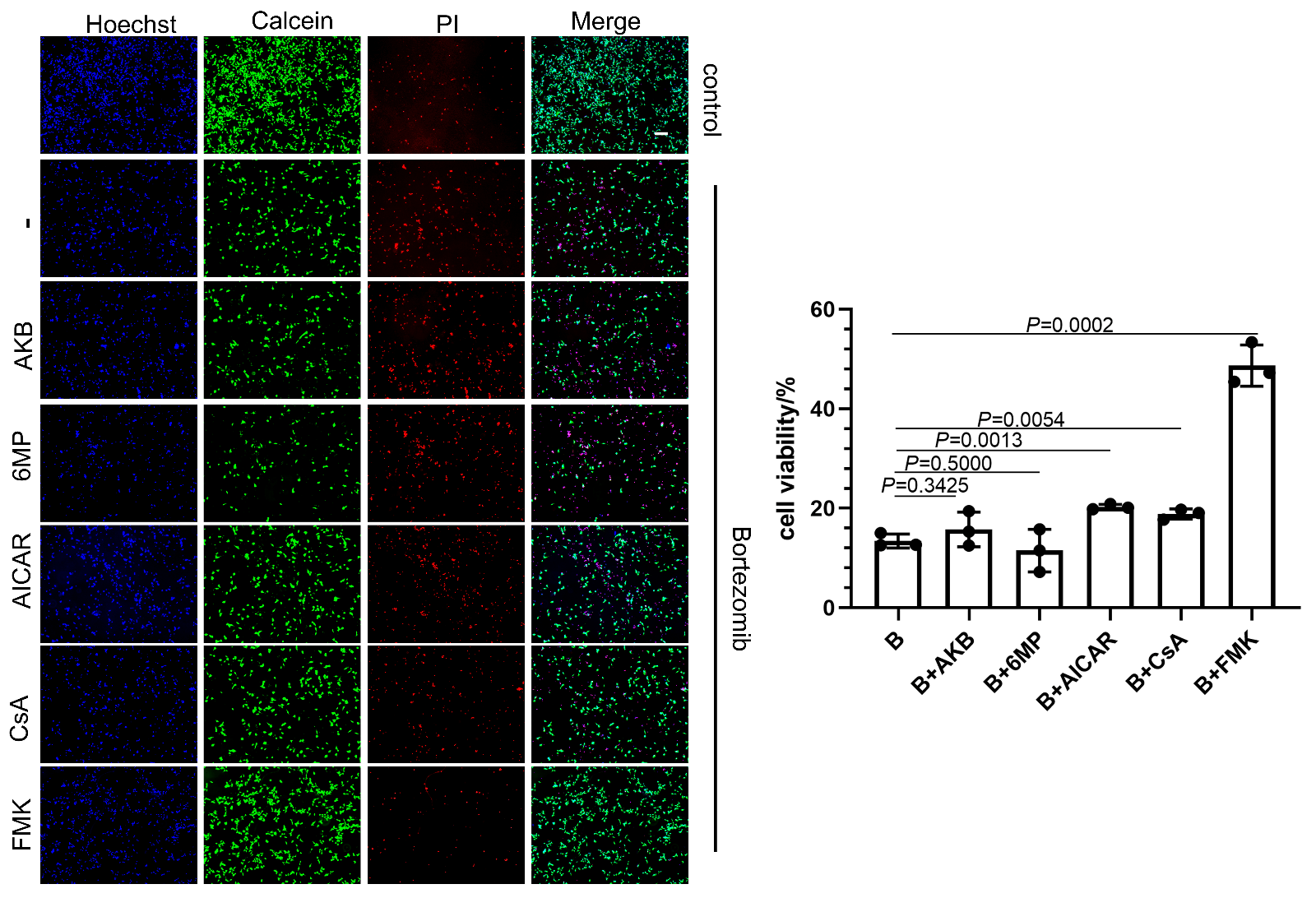


**Figure S14.** Viability of cells pretreated 2 h with 1 mM AKB, 100 μM 6-MP, 400 μM AICAR and 600 nM CsA, 50 μM FMK, then treated with 50 nM bortezomib (B) for 24 h (unpaired *t*-test). Means ± SD. n=3. Calcein fluorescence (green), PI (red), Hoechst 33342(blue). Scale bar, 100 μm.


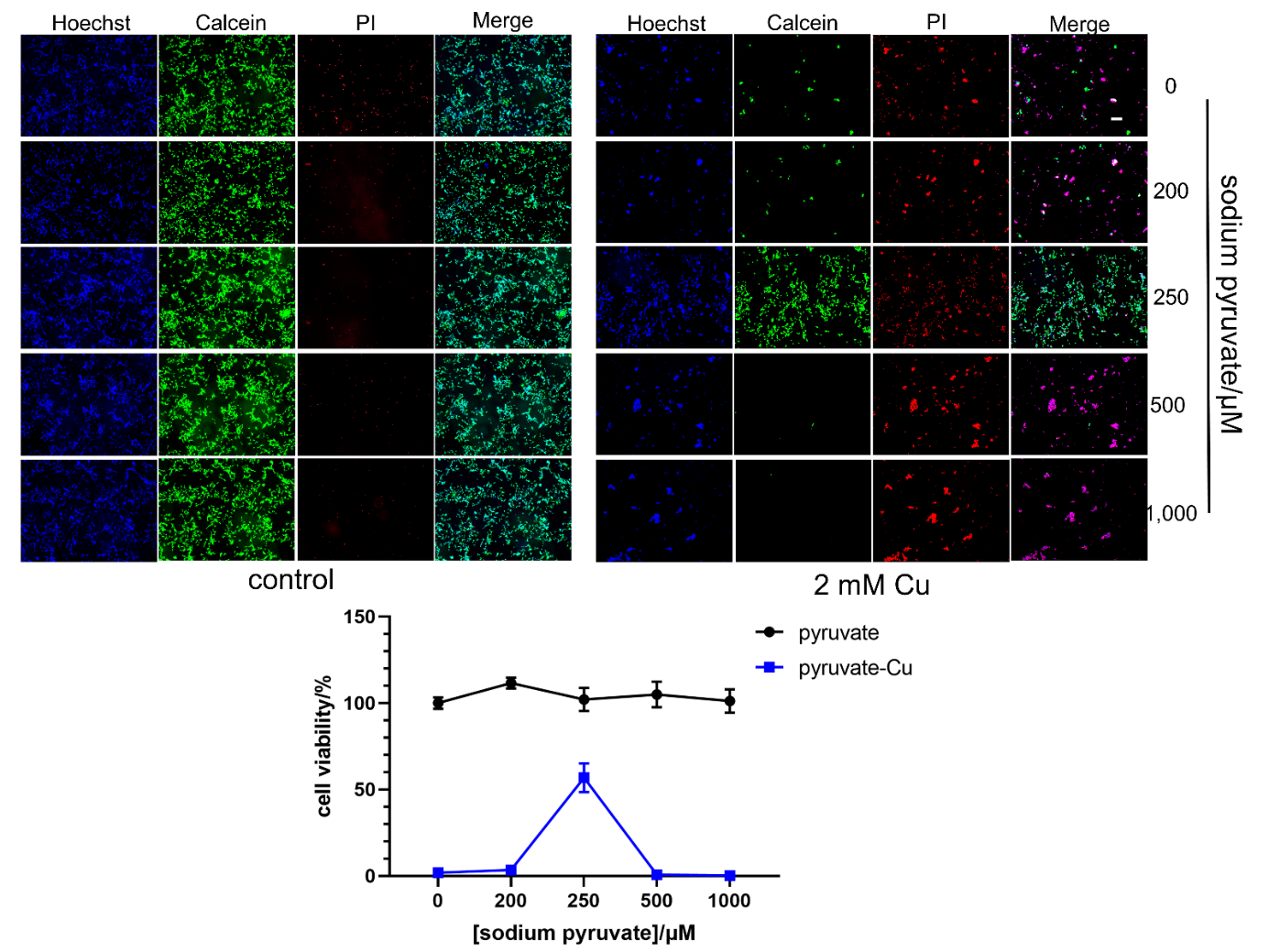
**Figure S15.** Viability of cells treated 6 h with sodium pyruvate and 2 mM CuSO_4_. Means ± SD. n=3. Calcein fluorescence (green), PI (red), Hoechst 33342(blue). Scale bar, 100 μm.
