# Supplementary material for: NADH‐Reductive Stress Induced by Dihydrolipoamide Dehydrogenase Activation Contributes to Cuproptosis
Source: Adv Sci (Weinh). 2025 Dec 5;13(9):e20444. doi: 10.1002/advs.202520444 (PMC12904017; doi:10.1002/advs.202520444)
Supplement: Supplementary file 2 — Supporting Information [file ADVS-13-e20444-s001.docx]

Supporting Information

**NADH-reductive stress induced by dihydrolipoamide dehydrogenase activation contributes to cuproptosis**

*Si-Yi Zhang, Xing-Hua Ren, Cheng-Hong Zhang, Zhan-You Wang**

(* for corresponding author)

Fig.1 C. DLAT and its oligomers





Fig.1 C. LIAS





Fig.1 C. FDX1





Fig.1 C. DLD





Fig.1 C. β-actin





Fig.1 I. DLAT and its oligomers





Fig.1 I. β-actin





Fig.4 H. p-AMPK





Fig.4 H. AMPK





Fig.4 H. β-actin
